# Delayed Demyelination and Impaired Remyelination in Aged Mice in the Cuprizone Model

**DOI:** 10.3390/cells9040945

**Published:** 2020-04-11

**Authors:** Stefan Gingele, Florian Henkel, Sandra Heckers, Thiemo M. Moellenkamp, Martin W. Hümmert, Thomas Skripuletz, Martin Stangel, Viktoria Gudi

**Affiliations:** Department of Neurology, Hannover Medical School, D-30625 Hannover, Germany; gingele.stefan@mh-hannover.de (S.G.); Florian.Henkel@stud.mh-hannover.de (F.H.); sandra.heckers@gmx.de (S.H.); thiemo.m.moellenkamp@stud.mh-hannover.de (T.M.M.); huemmert.martin@mh-hannover.de (M.W.H.); skripuletz.thomas@mh-hannover.de (T.S.); stangel.martin@mh-hannover.de (M.S.)

**Keywords:** multiple sclerosis, cuprizone, age, oligodendrocyte, myelin, demyelination, remyelination, microglia, astrocyte

## Abstract

To unravel the failure of remyelination in multiple sclerosis (MS) and to test promising remyelinating treatments, suitable animal models like the well-established cuprizone model are required. However, this model is only standardized in young mice. This does not represent the typical age of MS patients. Furthermore, remyelination is very fast in young mice, hindering the examination of effects of remyelination-promoting agents. Thus, there is the need for a better animal model to study remyelination. We therefore aimed to establish the cuprizone model in aged mice. 6-month-old C57BL6 mice were fed with different concentrations of cuprizone (0.2–0.6%) for 5–6.5 weeks. De- and remyelination in the medial and lateral parts of the corpus callosum were analyzed by immunohistochemistry. Feeding aged mice 0.4% cuprizone for 6.5 weeks resulted in the best and most reliable administration scheme with virtually complete demyelination of the corpus callosum. This was accompanied by a strong accumulation of microglia and near absolute loss of mature oligodendrocytes. Subsequent remyelination was initially robust but remained incomplete. The remyelination process in mature adult mice better represents the age of MS patients and offers a better model for the examination of regenerative therapies.

## 1. Introduction

Multiple sclerosis (MS) is a chronic inflammatory disease of the central nervous system (CNS). Destruction and loss of oligodendrocytes resulting in demyelination represent the pathological core characteristics of MS [1]. Remyelination is a naturally occurring and highly effective repair mechanism after demyelination that can restore rapid axonal conduction velocity [2] and lead to functional recovery and neuroprotection [3,4,5]. It was suggested that remyelination might prevent axonal injury and chronic clinical disability [6,7]. However, remyelination varies considerably between individual patients and lesion location and is often incomplete or fails, particularly in chronic MS lesions [8,9]. To decipher the pathophysiology of remyelination and the reasons for its failure as well as to investigate remyelination-supporting agents, toxic demyelination animal models such as the cuprizone mouse model are needed. In the cuprizone model, usually young mice are fed with the copper chelator cuprizone (bis-cyclohexanone oxaldihydrazone) which leads to highly reproducible oligodendrocyte apoptosis followed by demyelination. Typically, C57BL6 mice aged 8–10 weeks are fed a diet containing 0.2% cuprizone for 5 weeks to induce complete demyelination of the midline of the corpus callosum (CC). After withdrawal of cuprizone, robust remyelination occurs within days [10,11,12]. However, this standard protocol of cuprizone intoxication does not reflect the complex situation in MS lesions with limited remyelination capacity. Furthermore, myelin repair occurs rapidly, which causes difficulties for the investigation of remyelination-promoting therapeutic approaches. In addition, the age of 8–10 weeks in mice is equivalent to a human age below 18 years, rather representing the situation in pediatric MS [13,14,15]. In contrast, an age of 6 months in C57BL6 mice represents a mature adult phenotype and corresponds better to the mean age of MS disease onset in humans of approximately 30 years [13,14,15]. Additionally, remyelination efficiency shows an age related decline in different toxic demyelination models and thus investigation of this regenerative process in aged animals represents a promising approach to gain insights into impediments of remyelination [16,17,18,19,20]. Although the cuprizone model has previously been used in aged mice, the protocols differed considerably regarding the age, the concentration, and duration of cuprizone treatment [21,22,23,24]. However, it is crucial to exactly determine the cuprizone dose and feeding period to achieve complete and reproducible demyelination of the region of interest to investigate subsequent remyelination [12,25,26]. Thus, we established a protocol to induce complete demyelination of the corpus callosum in 6-month-old male C57BL6 mice and characterized the de- and remyelination processes in detail.

## 2. Materials and Methods

### 2.1. Animals

Male C57BL6/J mice were purchased from Charles River Laboratories (Sulzfeld, Germany). Animals underwent routine cage maintenance once a week and were microbiologically monitored according to the Federation of European Laboratory Animal Science Associations recommendations [27]. Food and water were available ad libitum. All research procedures were approved by the Review Board for the Care of Animal Subjects of the district government (LAVES, Lower Saxony, Germany; ethic approval number: 15/1762) and performed according to international guidelines on the use of laboratory animals.

### 2.2. Induction of De- and Remyelination

In order to establish the cuprizone treatment protocol for aged mice representing a mature adult phenotype [13] and resulting in complete demyelination of the midline of the corpus callosum, 6-month-old male C57BL6/J mice were fed with different concentrations (0.2%, 0.3%, 0.4%, 0.5%, or 0.6%) of cuprizone (bis-cyclohexanone oxaldihydrazone, Sigma-Aldrich, St. Louis, MO, USA) mixed into a milled standard rodent chow (maintenance diet, rats/mice, Altromin, Lage, Germany). For induction of experimental demyelination, cuprizone was administered for up to 6.5 weeks. In total, demyelination in aged mice was studied in 12 different treatment groups. After cuprizone feeding, mice were changed to standard rodent chow and subsequent remyelination was assessed at different time points (up to 3.5 weeks after the end of cuprizone feeding) (see Figure S1 for experimental setup). Age-matched male C57BL6/J control mice received standard rodent chow without cuprizone. Toxic demyelination in young mice was induced as previously described [28] by feeding 2-month-old male C57BL6/J mice a diet containing 0.2% cuprizone for 5 weeks. After 5 weeks, cuprizone was removed from the diet and remyelination was assessed after another 1.5 weeks (Figure S1). Five to six animals were analyzed for each cuprizone concentration and every time point during de- and remyelination in young and aged mice.

### 2.3. Tissue Processing

Mice were perfused at different time points with 4% paraformaldehyde in phosphate buffer through the left cardiac ventricle as previously described [29,30]. The brains were removed, post-fixed in 4% paraformaldehyde and embedded in paraffin. For light microscopy, 7 µm serial coronal paraffin sections between bregma −0.82 mm and bregma −1.94 mm according to the mouse atlas by Paxinos and Franklin [31] were cut with a rotary microtome (RM2245, Leica, Wetzlar, Germany) and evaluated microscopically.

### 2.4. Immunohistochemistry

Paraffin-embedded sections were dewaxed and heat-unmasked in 10 mM citrate buffer (pH 6.0). The following primary antibodies were used for immunostaining: for myelin, anti-myelin basic protein (MBP, mouse monoclonal IgG2a, 1:500, BioLegend, San Diego, CA, USA) and anti-proteolipid protein (PLP, mouse monoclonal IgG2a, 1:500, BIO-RAD); for microglia, anti-ionized calcium-binding adaptor molecule 1 (Iba-1, rabbit polyclonal IgG, 1:200, Wako, Osaka, Japan) and for activated microglia Ricinus communis antigen 1 (RCA-1, 1:1000, Vector); for mature oligodendrocytes anti-adenomatous polyposis coli (APC, mouse IgG2b, 1:200, Calbiochem) and anti-myelin-associated neurite outgrowth inhibitor (NOGO-A, rabbit polyclonal IgG, 1:750, Millipore, Burlington, MA, USA); for astrocytes, anti-glial fibrillary acidic protein (GFAP, polyclonal rabbit IgG, 1:200, Dako, Santa Clara, CA, USA); for axonal damage anti-amyloid precursor protein (APP, rabbit polyclonal IgG, 1:200, Serotec, Hercules, CA, USA) and anti-Synaptophysin (mouse monoclonal IgG1, 1:200, BIO-RAD, Hercules, CA, USA). All antibodies were diluted in PBS containing 0.3% Triton-X100. Sections were further incubated with biotinylated secondary antibodies, followed by peroxidase-coupled avidin-biotin complex (ABC Kit, Vector Laboratories, Burlingame, CA, USA). For immunofluorescence double staining, sections were incubated with secondary antibodies Alexa Fluor 555 goat anti-rabbit IgG (H + L) and Alexa Fluor 488 goat anti-mouse IgG (H + L) (all 1:500, Invitrogen, Carlsbad, CA, USA). Brain slices were counterstained either with Mayer’s hemalum solution (Merck, Darmstadt, Germany) for DAB-based cell analysis or with 4′,6-diamidino-2-phenylindole (DAPI, Invitrogen, Carlsbad, CA, USA) for immunofluorescence staining.

### 2.5. Determination of Demyelination and Quantification of Glial Reaction

The extent of cuprizone-induced demyelination was assessed as previously described [32,33]. Sections immunostained for myelin proteins were scored by three independent observers using a magnification of 200× in the midline of the corpus callosum and in the lateral parts directly adjacent to the midline using a light microscope (Olympus BX61, Olympus, Hamburg, Germany). On a scale from 0 (complete loss of myelin staining), to 3 (normal, fully myelinated corpus callosum) the different grades of demyelination were assessed (see [34] for representative images of the respective grades). In this grading system, a score of 1 is equivalent to one third myelinated area and a score of 2 corresponds to two thirds myelinated area of the investigated region of the corpus callosum, respectively [35]. Control tissues did not display any abnormalities. To quantify the reactions of different glial cell populations, immunopositive cells were counted at a magnification of 400× (Olympus BX61, Olympus cellSens Software) in the midline and in the flanking lateral parts of the corpus callosum. Results of cell counting refer to the number of cells per mm². Synaptophysin/APP double positive spheroids in the size >2 µm were counted in the central corpus callosum using a magnification of 400× (Olympus BX61, Olympus cellSens Software). The results are presented as mean with standard error of the mean (SEM) of spheroid numbers per mm^2^.

### 2.6. Statistical Analysis

Normal distribution was evaluated by using Kolmogorov-Smirnov test and statistical analysis was performed using analysis of variance one-way ANOVA followed by Bonferroni´s multiple comparison test or Kruskal-Wallis test followed by Dunn’s multiple comparison test when appropriate.

All data are given as arithmetic means ± standard error of the mean (SEM). Significant effects are indicated by asterisks (compared to the prior time point) or hash marks (compared to controls) (*/^#^ *p* < 0.05; **/^##^ *p* < 0.01; ***/^###^ *p* < 0.001) and are shown in the respective figures.

## 3. Results

### 3.1. The Standard 0.2% Cuprizone Treatment is Not Sufficient to Induce Demyelination of the Corpus Callosum in Aged Mice

**Figure 1:**
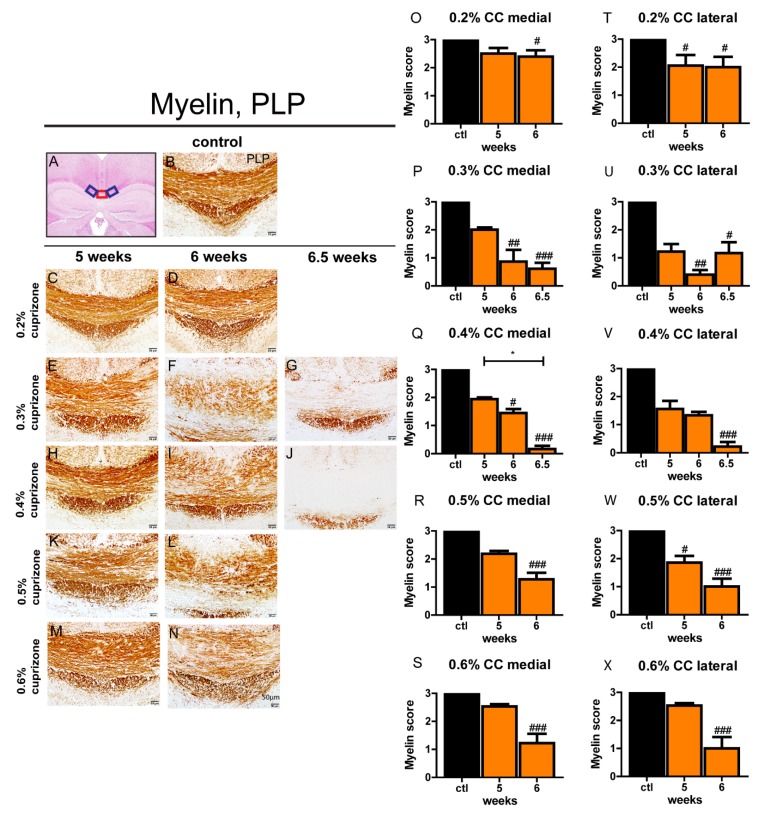
PLP loss during demyelination in the corpus callosum of aged mice. Hematoxylin and eosin (H & E)-stained control mouse brain (**A**) shows the areas of the corpus callosum that were analyzed (red box: medial part; blue boxes: lateral parts). Representative PLP-stained sections (**C**–**N**) show a decrease of PLP-positive myelin fibers during the course of demyelination in the central corpus callosum of aged mice after cuprizone treatment with different concentrations (0.2–0.6% cuprizone) for different feeding periods (5, 6, or 6.5 weeks). An exemplary picture (**B**) shows PLP staining in the midline of the corpus callosum of an age-matched control animal. A myelination score of 3 represents complete myelination, whereas a score of 0 represents complete demyelination. Graphs display the myelin score per time point and group in the midline (**O**–**S**) and in the lateral part of the corpus callosum (**T**–**X**) in aged animals. Bars represent mean + SEM. Significant effects between different investigated time points are indicated by asterisks and effects in comparison to control are indicated by hashmarks (*/^#^ *p* < 0.05; **/^##^ *p* < 0.01; ***/^###^ *p* < 0.001). Ctl. = control; 5, 6, or 6.5 weeks = treatment period with cuprizone for respective duration. N = 5–6 animals per group.

To establish a cuprizone treatment protocol in mature adult mice, we first followed the feeding protocols in young mice and fed 6-month-old male C57BL6/J mice a ground standard rodent chow containing 0.2% cuprizone for 5 or 6 weeks. To determine demyelination in aged mice, the midline and lateral parts of the corpus callosum in MBP and PLP-stained sections were investigated. The commonly established feeding protocol for young mice (5 weeks, 0.2% cuprizone) was not sufficient to induce significant loss of MBP or PLP myelin protein in both the medial and lateral parts of the corpus callosum of aged mice (Figure 1 and Figure S2). Moreover, a prolonged treatment period of 6 weeks 0.2% cuprizone did not lead to significant demyelination (Figure 1 and Figure S2).

### 3.2. Feeding of 0.4% Cuprizone for 6.5 Weeks Results in Complete Demyelination of the Corpus Callosum in Aged Mice

An increase of cuprizone dose to 0.3–0.6% for 5 weeks resulted in a significant but nonetheless incomplete reduction of MBP and PLP in the midline of the corpus callosum (Figure 1 and Figure S2). After an extended feeding period of cuprizone for 6 weeks, nearly complete loss of MBP protein in the medial and lateral parts of the corpus callosum was evident (Figure S2), whereas PLP immunoreactivity was only partially reduced in the midline and adjacent lateral sections of the corpus callosum (Figure 1). Since demyelination in the midline of the corpus callosum was most pronounced after treatment with 0.3% and 0.4% cuprizone for 6 weeks with no dose-dependent advancement of demyelination by feeding higher concentrations of cuprizone, we prolonged the cuprizone treatment with 0.3% and 0.4% for an additional 3 days to 6.5 weeks in total to achieve complete demyelination. As expected, 0.4% cuprizone feeding for 6.5 weeks resulted in virtually complete demyelination of the midline and adjacent parts of the corpus callosum as judged by distinct loss of MBP and PLP immunoreactivity (Figure 1 and Figure S2). In contrast, after treatment with 0.3% cuprizone for 6.5 weeks, demyelination was insufficient (Figure 1 and Figure S2) and re-expression of myelin proteins, especially of MBP, was already evident in the corpus callosum (Figure S2). Interestingly, demyelination in the lateral segments of the corpus callosum generally preceded demyelination of the medial part of the corpus callosum (Figure S2).

### 3.3. Cuprizone Treatment Leads to Significant Oligodendrocyte Loss Independent of Cuprizone Concentration

**Figure 2:**
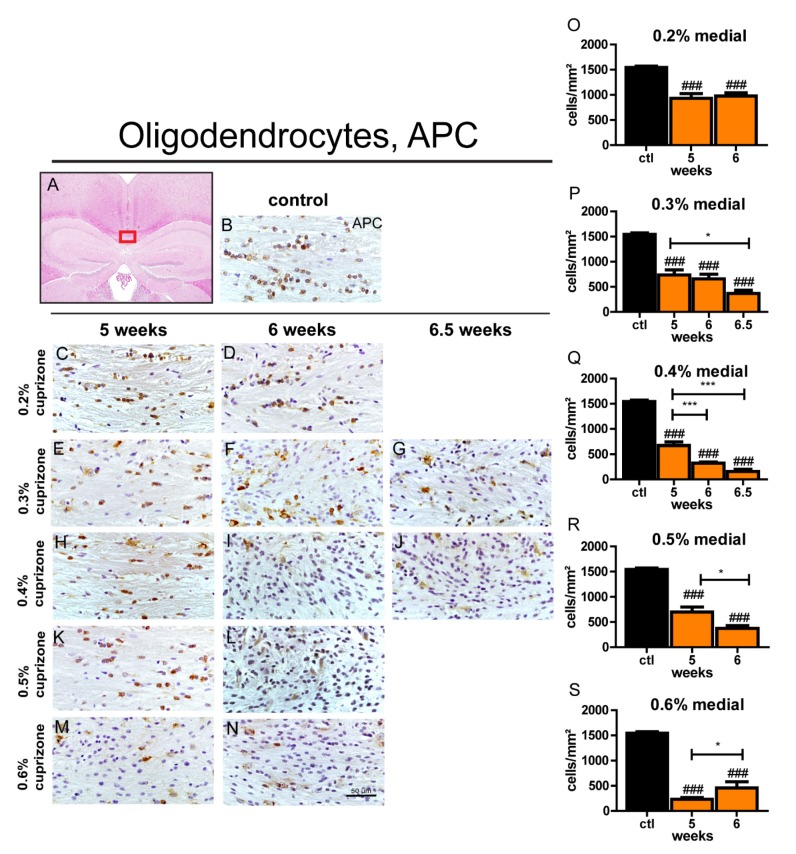
Depletion of adult oligodendrocytes in the midline of the corpus callosum in aged mice after different cuprizone feeding protocols. The outlined area (red box) shows the medial part of the corpus callosum which was investigated (**A**). Representative images from APC-stained sections (**C**–**N**) show the depletion of oligodendrocytes during the course of demyelination in the midline of the corpus callosum of aged mice after cuprizone treatment with different concentrations (0.2–0.6% cuprizone) for different time periods (5, 6, or 6.5 weeks). (**B**) shows a control brain section stained with APC. Graphs (**O**–**S**) represent numbers of APC-positive cells/mm^2^ in the medial part of the corpus callosum in aged animals. Bars display mean + SEM. Significant effects between different investigated time points are indicated by asterisks and effects in comparison to control are indicated by hashmarks (*/^#^ *p* < 0.05; **/^##^ *p* < 0.01; ***/^###^ *p* < 0.001). Ctl. = control; 5, 6, or 6.5 weeks = feeding period of cuprizone for respective duration. N = 5–6 animals per group.

To evaluate how mature oligodendrocytes were affected by different cuprizone feeding protocols, the oligodendrocyte markers APC and Nogo-A were examined in the corpus callosum. Each cuprizone concentration and feeding duration was sufficient to significantly deplete oligodendrocytes as compared to controls (Figure 2 and Figure S3). However, the reduction of APC and Nogo-A-positive cells in the corpus callosum of aged mice was less pronounced after feeding 0.2% cuprizone, as used as standard protocol for young mice compared to higher concentrations of 0.3–0.6%. This result is of particular interest as it demonstrates that treatment with 0.2% cuprizone is sufficient to significantly damage mature oligodendrocytes in aged mice without inducing relevant demyelination, as mentioned before. Feeding higher doses of 0.3–0.6% cuprizone resulted in more distinctive depletion of mature oligodendrocytes with the most extensive loss of adult oligodendrocytes in the midline of the corpus callosum being observed after feeding 0.4% cuprizone for 6.5 weeks (Figure 2 and Figure S3). Again, in line with the findings from the myelin stainings, a dose-dependent effect on oligodendrocyte loss appeared to exist up to a concentration of 0.4% and higher concentrations of cuprizone did not result in faster or more pronounced oligodendrocyte damage. Rather, a duration-dependent effect was observed with feeding durations of 6 and 6.5 weeks being more effective than 5 weeks for concentrations between 0.3% and 0.5% cuprizone (Figure 2). Similar to the medial part, pronounced oligodendrocyte depletion was detected in the lateral segments of the corpus callosum as well (Figure S5).

### 3.4. Maximum Microglia Activation and Accumulation is Evident after 6.5 Weeks with 0.4% Cuprizone Treatment

**Figure 3:**
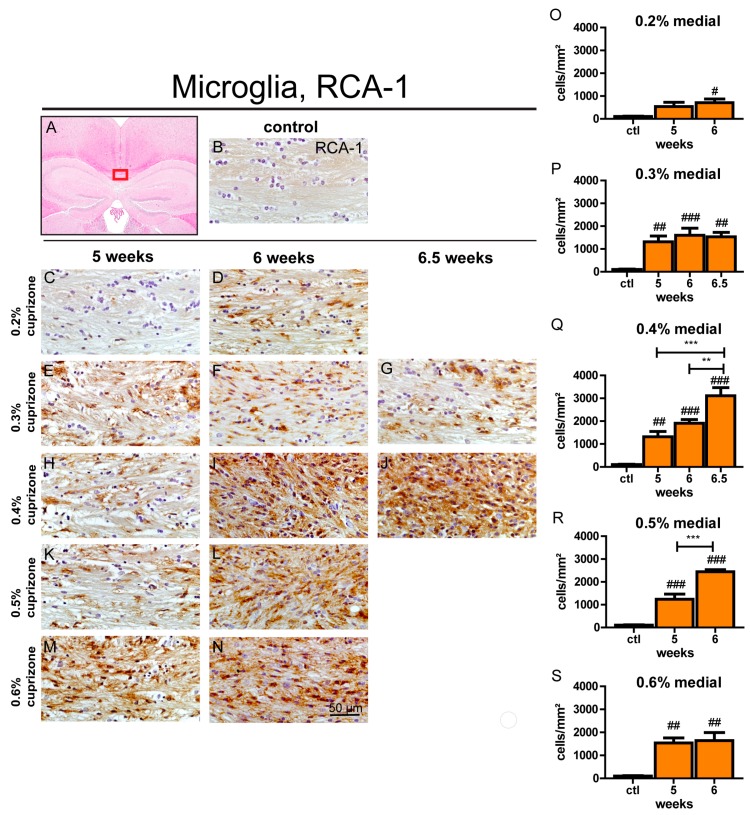
Activation of microglia in the central corpus callosum during cuprizone-induced demyelination in aged mice. The red box in (**A**) displays the medial section of the corpus callosum that was analyzed. Representative pictures (**C**–**N**) of RCA-1-stained brain sections show accumulation of activated microglia during the course of demyelination in the midline of the corpus callosum of aged mice after cuprizone treatment with various doses (0.2–0.6% cuprizone) for different feeding periods (5, 6, or 6.5 weeks). An exemplary brain section of a control animal stained for RCA-1 is shown in (**B**). Graphs depict the amount of RCA-1-positive cells/mm^2^ per time point in the midline of the corpus callosum in aged animals (**O**–**S**). Bars represent mean + SEM. Effects between different investigated time points are indicated by asterisks and effects in comparison to control are indicated by hash marks (*/^#^ *p* < 0.05; **/^##^ *p* < 0.01; ***/^###^ *p* < 0.001). Ctl. = control; 5, 6, or 6.5 weeks = feeding period of cuprizone for respective duration. N = 5–6 animals per group.

To assess microglia reaction during cuprizone-induced demyelination in aged mice, the number of activated microglia was determined by RCA-1 staining. Iba-1 was used to quantify accumulation of the entire microglia population. Applying the feeding protocol of young mice with 0.2% cuprizone for 5 weeks resulted in a significant infiltration of Iba-1-positive cells (Figure S4) but only mild and not significant activation of these microglia was apparent compared to controls (Figure 3). Prolongation of cuprizone treatment to 6 weeks led to more pronounced activation of microglial cells in the midline of the corpus callosum (Figure 3). An increase of cuprizone dose (0.3–0.6%) led to a robust accumulation and activation of microglia already after 5 weeks of cuprizone feeding, and extending cuprizone treatment for an additional week enhanced these effects in most treatment groups (Figure 3 and Figure S4). Interestingly, the effect of dose increase between the 0.3% and 0.6% conditions on the amount and activation of microglia was limited. The maximum microglial activation and accumulation in the midline of the corpus callosum as judged by RCA-1 and Iba-1 immunopositive cells was actually seen after feeding 0.4% cuprizone for 6.5 weeks. In contrast, prolonging 0.3% cuprizone treatment to 6.5 weeks did not result in increased microglia activation or cell count (Figure 3 and Figure S4). The lateral parts of the corpus callosum exhibited a more pronounced increase of activated microglia after just 5 weeks of cuprizone feeding as compared to the medial part (Figure S5). Similar to the midline of the corpus callosum, there was no clear impact of increasing cuprizone concentrations on induction of microglia reaction, with feeding protocols containing 0.5% and 0.6% cuprizone even showing a tendency towards less pronounced microglial accumulation. The maximum amount of RCA-1 and Iba-1-positive cells in the lateral segments was also observed after feeding 0.4% cuprizone for 6.5 weeks (Figure S5).

### 3.5. The Extent of Astrocytosis is Independent of Duration and Dose of Cuprizone Treatment

**Figure 4:**
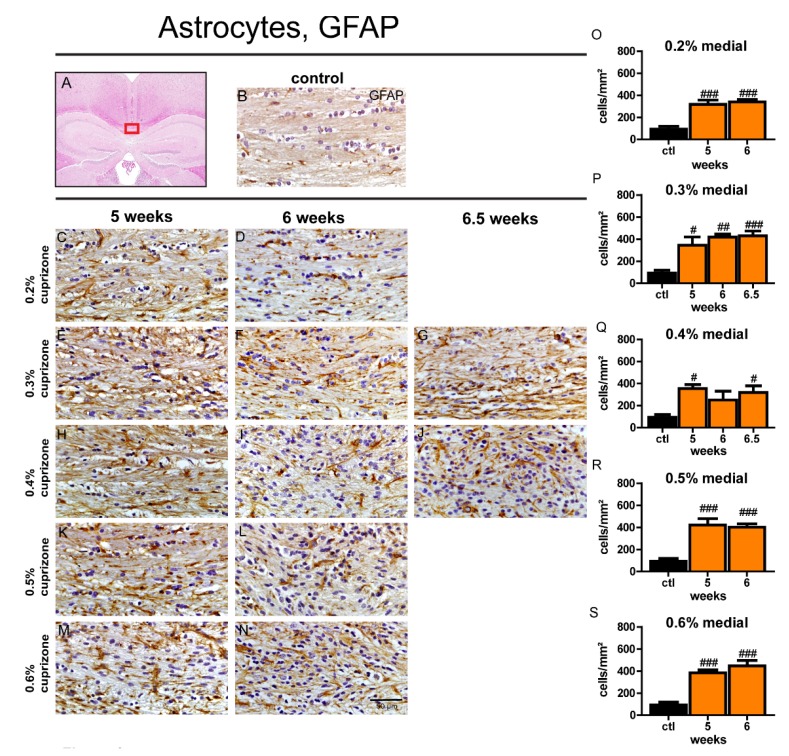
Astrocytosis in the medial corpus callosum of aged mice after different cuprizone treatment protocols. The boxed area represents the medial part of the corpus callosum which was examined (**A**). An exemplary picture shows a brain section of a control animal stained for GFAP (**B**). Representative images of GFAP-stained sections (**C**–**N**) depict astrocyte hypertrophy and hyperplasia during demyelination in the central corpus callosum of aged mice after cuprizone treatment with different concentrations (0.2–0.6% cuprizone) for different treatment periods (5, 6, or 6.5 weeks). Graphs show the number of GFAP-positive astrocytes/mm^2^ in the midline of the corpus callosum in aged animals (**O**–**S**). Bars represent mean + SEM. Effects between different investigated time points are indicated by asterisks and effects in comparison to control are indicated by hashmarks (*/^#^ *p* < 0.05; **/^##^ *p* < 0.01; ***/^###^ *p* < 0.001). Ctl. = control; 5, 6, or 6.5 weeks = feeding period of cuprizone for respective duration. N = 5–6 animals per group.

The astrocyte response to cuprizone-induced demyelination in the corpus callosum of aged mice was examined by staining for GFAP. Feeding of cuprizone resulted in significantly increased numbers of GFAP-positive astrocytes in the midline and adjacent lateral segments of the corpus callosum in virtually all treatment groups compared to control animals (Figure 4 and Figure S5). No significant changes of the amount of GFAP-expressing cells were observed between the different treatment groups depending on feeding duration or cuprizone concentration. Similar trends in the astrocyte reaction were observed in the lateral part compared to the medial part of the corpus callosum (Figure S5).

### 3.6. Aged Mice Show Rapid Induction of Remyelination but Remyelination Remains Incomplete

**Figure 5:**
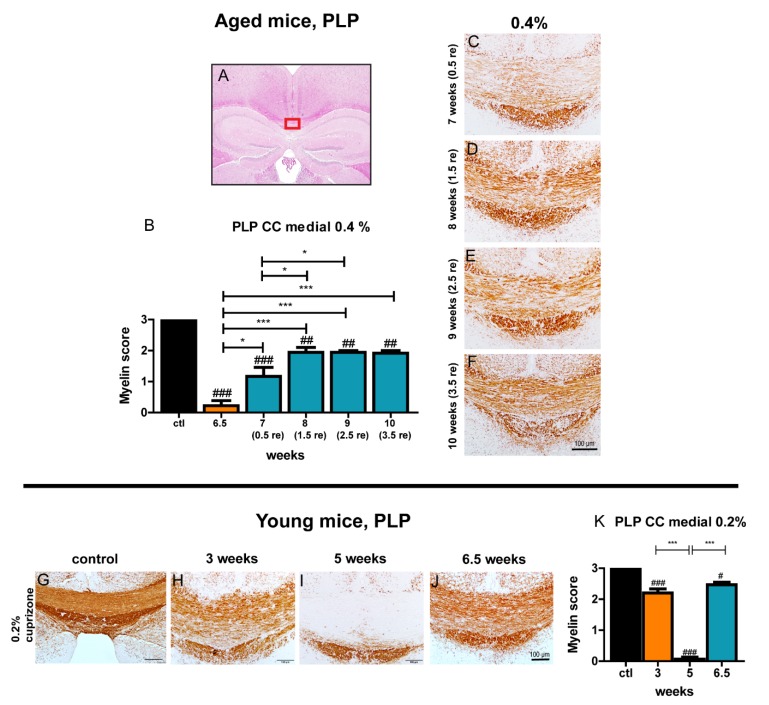
The marked area (red box) shows the medial part of the corpus callosum which was examined (**A**). (**B**) shows the myelin score for PLP in the midline of the corpus callosum in aged animals fed with 0.4% cuprizone for 6.5 weeks and subsequent remyelination (0.5, 1.5, 2.5, and 3.5 weeks after cessation of treatment with cuprizone). A score of 3 represents complete myelination, whereas a score of 0 represents complete demyelination. Representative PLP-stained sections (**C**–**F**) demonstrate the course of remyelination in aged mice. See Figure 1 for a representative image of a control animal and of demyelination after 0.4% cuprizone feeding for 6.5 weeks in aged mice. The myelin score for young mice treated with 0.2% cuprizone for 5 weeks and subsequent remyelination for 1.5 weeks is shown in **K**. Representative PLP-stained sections show the course of de- and remyelination in young mice (**G**–**J**). Bars display mean + SEM. Significant effects between different investigated time points are indicated by asterisks and effects in comparison to control are indicated by hashmarks (*/^#^ *p* < 0.05; **/^##^ *p* < 0.01; ***/^###^ *p* < 0.001). Ctl. = control; 6.5 weeks = feeding period of 0.4% cuprizone in aged mice; 7 (0.5 re) weeks = 0.5 weeks of remyelination, 8 (1.5 re) weeks = 1.5 weeks of remyelination, 9 (2.5 re) weeks = 2.5 weeks of remyelination, 10 (3.5 re) weeks = 3.5 weeks of remyelination in aged mice; 3 and 5 weeks = feeding period of 0.2% cuprizone in young mice; 6.5 (1.5) weeks = 1.5 weeks of remyelination in young mice. N = 5–6 animals per group.

After we established the best protocol for complete demyelination in the corpus callosum by feeding 0.4% cuprizone for 6.5 weeks, we investigated remyelination with this treatment regime. After removing cuprizone from the diet, remyelination was studied in the corpus callosum for up to 3.5 weeks. Remyelination in the medial and lateral parts of the corpus callosum was assessed by staining for the myelin proteins MBP and PLP. After the switch to normal chow, there was prompt and significant re-appearance of the myelin proteins PLP (Figure 5) and MBP (Figure S6) as early as 0.5 weeks after termination of cuprizone treatment (week 7). Interestingly, myelin staining with MBP remained unchanged after week 7 in the subsequently analyzed time points whereas PLP staining showed a further increase of myelin status at week 8 before reaching a plateau. Remarkably, even after 3.5 weeks of remyelination, expression of MBP and PLP myelin proteins did not reach the myelin status of control mice, but stagnated at a reduced level, representing partial myelination (Figure 5 and Figure S6). In contrast, young mice showed more advanced remyelination 1.5 weeks after termination of the cuprizone diet as visualized by MBP and PLP staining (Figure 5 and Figure S6) compared to aged mice. Remyelination in the lateral segments of the corpus callosum followed the same temporal pattern as in the midline area (Figure S9).

### 3.7. Repopulation of Oligodendrocytes in the Corpus Callosum during Remyelination is Less Efficient Compared to Young Mice

**Figure 6:**
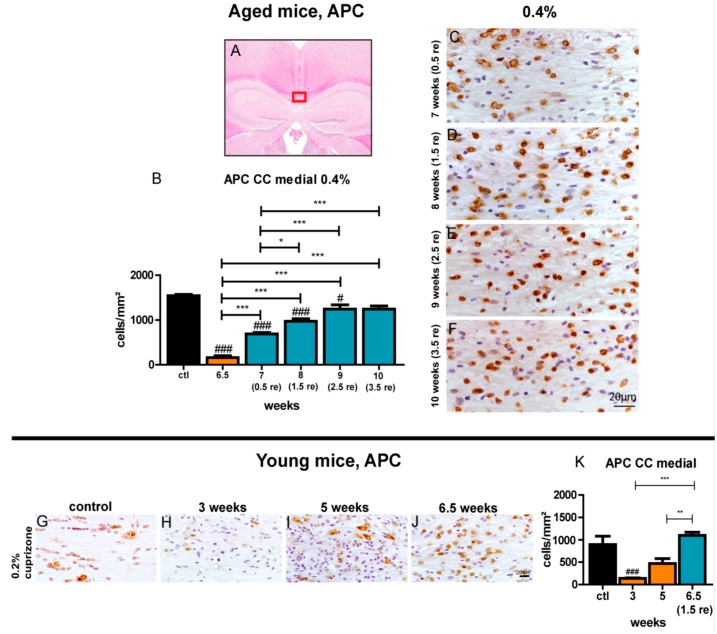
Repopulation of mature oligodendrocytes during remyelination in the medial corpus callosum of aged mice. The marked area (red box) in (**A**) shows the medial part of the corpus callosum that was investigated. Graphs display the number of APC-positive oligodendrocytes/mm^2^ for the respective time points of demyelination and subsequent remyelination in aged mice fed for 6.5 weeks with 0.4% cuprizone (**B**) and young mice treated for 5 weeks with 0.2% cuprizone (**K**). See Figure 2 for representative sections of aged mice for control and demyelination after 0.4% cuprizone feeding for 6.5 weeks, respectively. Exemplary images show repopulation of APC-positive oligodendrocytes during the course of remyelination in the central corpus callosum of aged mice (**C**–**F**) and during de- and remyelination in young mice (**G**–**J**). Bars show mean + SEM. Significant effects between different investigated time points are indicated by asterisks and effects in comparison to control are indicated by hashmarks (*/^#^ *p* < 0.05; **/^##^ *p* < 0.01; ***/^###^ *p* < 0.001). Ctl. = control; 6.5 weeks = feeding period of 0.4% cuprizone in aged mice; 7 (0.5 re) weeks = 0.5 weeks of remyelination, 8 (1.5 re) weeks = 1.5 weeks of remyelination, 9 (2.5 re) weeks = 2.5 weeks of remyelination, 10 (3.5 re) weeks = 3.5 weeks of remyelination in aged mice; 3 and 5 weeks = feeding period of 0.2% cuprizone in young mice; 6.5 (1.5) weeks = 1.5 weeks of remyelination in young mice. N = 5–6 animals per group.

APC and Nogo-A, as markers for mature oligodendrocytes, were used to investigate the reappearance of oligodendrocytes in the corpus callosum after demyelination induced by feeding mice with 0.4% cuprizone for 6.5 weeks. A significant increase in the number of APC- and Nogo-A-positive cells in the corpus callosum of aged mice was already detected 0.5 weeks after cessation of cuprizone feeding at week 7 (Figure 6 and Figure S7). The density of oligodendrocytes further grew steadily during the remyelination observation period. After 3.5 weeks of remyelination, the amount of adult oligodendrocytes in the midline of the corpus callosum almost equaled the control level. In contrast, in young mice, the density of Nogo-A- and APC-positive cells reached the level of control animals at the latest after 1.5 weeks of remyelination with a trend of even exceeding baseline numbers of controls (Figure 6 and Figure S7). Thus, repopulation of the demyelinated corpus callosum with mature oligodendrocytes seemed to proceed more slowly in aged mice compared to young animals. It is noteworthy that the absolute amount of oligodendrocytes in the corpus callosum of aged mice was higher compared to young mice in the control group (Figure 6 and Figure S7). Re-appearance of mature oligodendrocytes in the lateral parts of the corpus callosum occurred in a similar manner to that of the midline, though the number of Nogo-A-positive cells did not reach the cell count in control mice (Figure S9).

### 3.8. Prolonged Activation of Microglia during the Recovery Period

**Figure 7:**
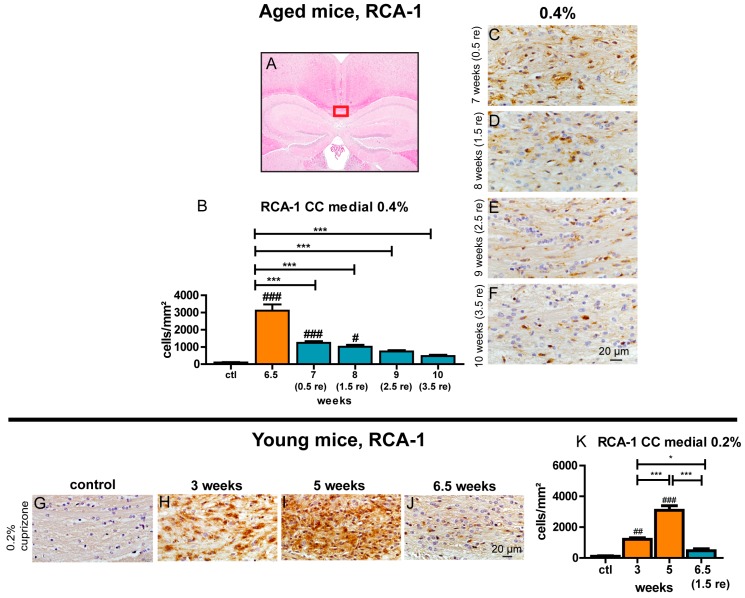
Delayed decrease of activated microglia during the course of remyelination in the medial corpus callosum of aged mice. The medial part of the corpus callosum that was analyzed is highlighted (**A**). Graphs show the amount of RCA-1-positive activated microglia/mm^2^ for the respective time points of demyelination and subsequent remyelination in aged mice treated with 0.4% cuprizone for 6.5 weeks (**B**) and young mice fed with 0.2% cuprizone for 5 weeks (**K**). See Figure 3 for representative images of RCA-1 staining of aged mice for controls and demyelination after 0.4% cuprizone feeding for 6.5 weeks, respectively. Representative sections show the decrease of activated microglia populations during the course of remyelination in the central corpus callosum of aged mice (**C**–**F**) and the increase of activated microglia during demyelination in young mice plus a decrease during subsequent remyelination (**G**–**J**). Bars show mean + SEM. Significant effects between different investigated time points are indicated by asterisks and effects in comparison to control are indicated by hashmarks (*/^#^ *p* < 0.05; **/^##^ *p* < 0.01; ***/^###^ *p* < 0.001). Ctl. = control; 6.5 weeks = feeding period of 0.4% cuprizone in aged mice; 7 (0.5 re) weeks = 0.5 weeks of remyelination, 8 (1.5 re) weeks = 1.5 weeks of remyelination, 9 (2.5 re) weeks = 2.5 weeks of remyelination, 10 (3.5 re) weeks = 3.5 weeks of remyelination in aged mice; 3 and 5 weeks = feeding period of 0.2% cuprizone in young mice; 6.5 (1.5) weeks = 1.5 weeks of remyelination in young mice. N = 5–6 animals per group.

Iba-1 and RCA-1 were used to quantify the degree of microglia cell accumulation and their activation status during remyelination. After a sharp rise of the amount of activated microglia was observed at 6.5 weeks of 0.4% cuprizone treatment, a swift and pronounced decrease of the number of RCA-1-positive activated microglia was apparent within the first days after cessation of cuprizone treatment (Figure 7). Interestingly and in contrast to the fast return of the elevated RCA-1-positive cell count in young mice towards control levels during remyelination, significantly elevated numbers of activated microglia persisted until week 8, and a trend towards an enhanced amount of these cells was obvious until the end of the observation period at week 10. The overall Iba-1-positive microglia population followed a similar temporal pattern compared to RCA-1-positive activated microglia (Figure S8). Additionally, the lateral segments of the corpus callosum displayed the same changes of microglia during remyelination in comparison to the processes observed in the medial part of the corpus callosum (Figure S9).

### 3.9. Astrogliosis Remains Unchanged Despite Ongoing Remyelination

**Figure 8:**
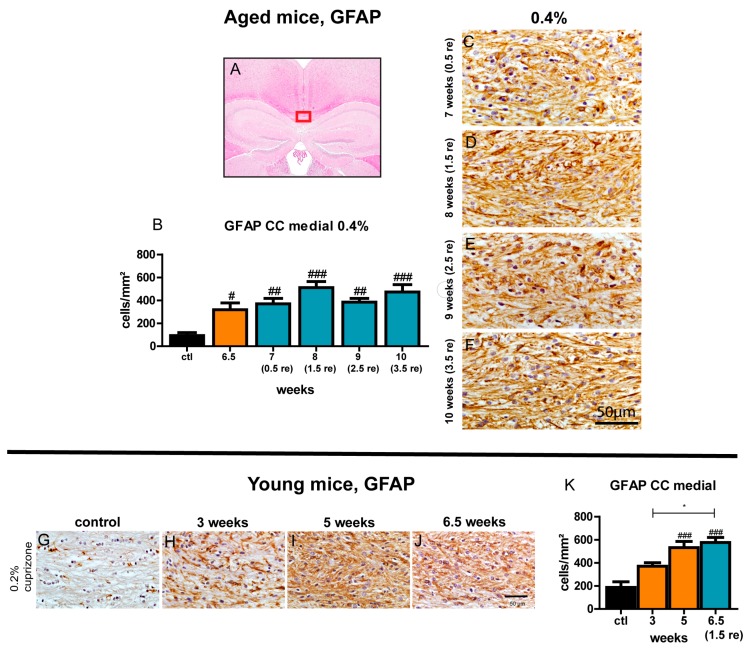
Persisting astrocytosis during remyelination in the midline of the corpus callosum of aged and young mice. The medial part of the corpus callosum that was investigated is marked (**A**). Graphs display the quantity of GFAP-positive astrocytes/mm^2^ for the particular time points of demyelination and following remyelination in aged mice treated with 0.4% cuprizone for 6.5 weeks (**B**) and young mice fed with 0.2% cuprizone for 5 weeks (**K**). Representative sections of control and demyelination after 0.4% cuprizone feeding for 6.5 weeks in aged mice are shown in Figure 4. Exemplary sections show persistently elevated numbers of astrocytes during remyelination in aged mice (**C**–**F**) and during de- and remyelination in young mice (**G**–**J**). Bars show mean + SEM. Significant effects between different analyzed time points are indicated by asterisks and effects in comparison to control are indicated by hashmarks (*/^#^ *p* < 0.05; **/^##^ *p* < 0.01; ***/^###^ *p* < 0.001). Ctl. = control; 6.5 weeks = feeding period of 0.4% cuprizone in aged mice; 7 (0.5 re) weeks = 0.5 weeks of remyelination, 8 (1.5 re) weeks = 1.5 weeks of remyelination, 9 (2.5 re) weeks = 2.5 weeks of remyelination, 10 (3.5 re) weeks = 3.5 weeks of remyelination in aged mice; 3 and 5 weeks = feeding period of 0.2% cuprizone in young mice; 6.5 (1.5) weeks = 1.5 weeks of remyelination in young mice. N = 5–6 animals per group.

Staining for GFAP was used to determine astrocyte hypertrophy and hyperplasia during remyelination. At all investigated time points during the remyelination period, the number of GFAP-positive cells in the midline of the corpus callosum was significantly increased as compared to control (Figure 8). The extent of astrogliosis did not change significantly over the time course of remyelination and remained as high as at the point of maximum demyelination at 6.5 weeks. Astrocytosis in the lateral segments of the corpus callosum followed the same trend as in the medial segment of the corpus callosum (Figure S9).

### 3.10. Axonal Pathology Occurs during Cuprizone-Induced Demyelination in Aged Mice and Corresponds to Microglia Activation

**Figure 9:**
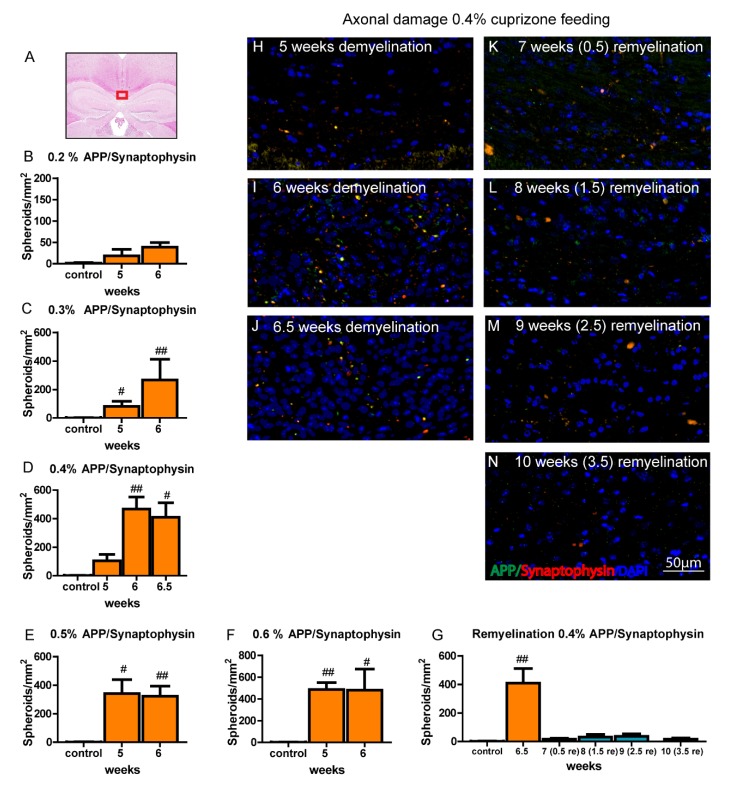
Axonal pathology during cuprizone-induced demyelination in aged mice. The medial part of the corpus callosum that was analyzed is highlighted (**A**). Graphs (**B**–**G**) represent number of APP/Synaptophysin double positive spheroids/mm^2^ per time point and group in aged mice during different cuprizone treatments protocols. Bars show mean + SEM. Significant effects between different analyzed time points are indicated by asterisks and effects in comparison to control are indicated by hashmarks (*/^#^ *p* < 0.05; **/^##^ *p* < 0.01; ***/^###^ *p* < 0.001). 6.5 weeks = feeding period of 0.4% cuprizone in aged mice; 7 (0.5 re) weeks = 0.5 weeks of remyelination, 8 (1.5 re) weeks = 1.5 weeks of remyelination, 9 (2.5 re) weeks = 2.5 weeks of remyelination, 10 (3.5 re) weeks = 3.5 weeks of remyelination in aged mice. N = 5–6 animals per group. Representative images show axonal damage/axonal transport disturbances during de- and remyelination in aged mice as depicted by the accumulation of pathological APP/Synaptophysin-positive spheroids in the central corpus callosum (**H**–**N**).

In order to evaluate axonal damage and vesicular axonal transport disturbances during cuprizone-induced demyelination in aged mice, the amount of APP/Synaptophysin double positive spheroids was determined in the medial section of the corpus callosum as previously described [28]. Similar to the reported changes in young mice, the number of APP/Synaptophysin-positive bulbs increased dramatically during cuprizone-induced demyelination, reaching a peak after 6–6.5 weeks of 0.4% cuprizone feeding (Figure 9) and corresponding to the peak of microglia accumulation and activation in this model (Figure 3 and Figure S4). During remyelination, the numbers of APP/Synaptophysin double positive beads rapidly decreased, however, larger-sized spheroids were still present in low numbers suggesting permanent axonal dissection (Figure 9). Standard treatment protocol with 5 weeks as well as a prolonged feeding period of 6 weeks with 0.2% cuprizone did not lead to significant axonal damage (Figure 9).

## 4. Discussion

Since remyelination is a highly effective regenerative process by which clinical disability can be prevented, development of remyelination-enhancing therapies for MS is an urgent medical need [36]. Therefore, the cuprizone mouse model represents a suitable approach to investigate remyelination failure and remyelination-promoting agents. However, the currently widely used cuprizone model is only well established in young (8–10 weeks old) mice displaying rapid and extensive remyelination [26] and thus does not reflect the situation of MS pathology in humans regarding age and remyelination efficiency. Therefore, we aimed to establish the cuprizone model in 6-month-old mice representing an aged, mature adult phenotype, corresponding to an adult age in humans in which MS is often diagnosed. Male C57BL6 mice were used since in these mice the cuprizone model is reliably implemented without interference of the hormonal cycle. The establishment of a standardized and optimized cuprizone treatment protocol for aged mice is particularly needed since widely varying cuprizone concentrations and feeding durations have been reported for aged mice with contradictory results [21,22,23,24].

We found that the standard cuprizone feeding protocol for young mice with 5 weeks 0.2% cuprizone was not sufficient to establish significant demyelination in aged mice. We show here that robust demyelination in aged mice is only up to a certain degree concentration-/dose-dependent and rather relies on a prolonged feeding duration, establishing 0.4% cuprizone for 6.5 weeks as the best cuprizone treatment protocol for mature adult (6 month) mice to achieve complete demyelination of the corpus callosum. This delayed progress of demyelination was not attributable to a single cell population. In contrast to young mice in which oligodendrocyte depletion and microglia accumulation precedes demyelination, in aged mice, the maximum degree of oligodendrocyte loss, microglia activation, and demyelination occurred simultaneously after 6.5 weeks of treatment with 0.4% cuprizone. This may be explained by a higher resistance of mature oligodendrocytes in aged animals against cuprizone-induced apoptosis on the one side and a reduced phagocytotic capacity of aged microglia resulting in delayed removal of myelin debris [18,37,38] on the other side. However, after demyelination was accomplished, prompt initiation of remyelination as judged by the re-expression of myelin markers accompanied by repopulation of the corpus callosum with oligodendrocytes was evident in aged mice. However, throughout the complete observation period of 3.5 weeks after withdrawal of cuprizone, myelination in the corpus callosum in aged mice remained diminished compared to control. This is in line with findings from ethidium bromide-induced focal demyelination in aged rats, in which remyelination was incomplete 4 weeks after lesion-induction [16]. A further increase in myelination over a longer remyelination period in our model is possible, however, there seemed to already be a stagnation of remyelination 1.5–2 weeks after cuprizone withdrawal. In accordance with remyelination remaining incomplete, re-appearance of mature oligodendrocytes in the corpus callosum was diminished and delayed compared to young mice. This finding is concordant with reports attributing the age-dependent decline of remyelination efficiency to a reduction of oligodendrocyte progenitor recruitment and differentiation [39]. Similar to the situation in young mice [28], axonal pathology was observed, accompanying the advancing degree of demyelination and microglia infiltration after 6 and 6.5 weeks of cuprizone treatment but rapidly improved during subsequent remyelination. Thus, this cuprizone protocol in aged mice displays important pathological hallmarks of human MS pathology [1].

Interestingly, after reaching a similar degree of activation and accumulation of microglia in the corpus callosum during demyelination compared to young mice, aged animals showed prolonged activation and an elevated cell count of microglia in the corpus callosum during the remyelination period. It is conceivable that this constitutes a reflection of the impaired ability of microglia in aged animals to resolve inflammation after demyelination [19]. One might speculate that microglia in aged mice do not represent a remyelination-supporting phenotype to the same extent as in young mice, e.g., by differently expressing pro-myelinating factors [40]. Furthermore, the phagocytosis activity may be diminished in aged microglia and thus may delay remyelination. Significant astrocytosis was observed throughout de- and remyelination and did not display significant changes over time. Since astrocytes are crucial for efficient remyelination after acute demyelination in young animals, e.g., by recruiting microglia and by producing pro-remyelinating factors [30,33], it stands to reason that astrocytes in aged mice are not as capable of creating a remyelination-supportive environment.

In summary, by comprehensively characterizing de- and remyelination in adult mature mice we established a reliable and feasible new protocol for the cuprizone model in which the remyelination capacity is impaired compared to young animals. This better represents the incomplete remyelination in human disease and allows for the study of the capacity of remyelinating agents.

## Supplementary Materials

The following are available online at https://www.mdpi.com/2073-4409/9/4/945/s1; Figure S1. Experimental design, Figure S2. MBP loss in the corpus callosum of aged mice after different cuprizone treatment protocols, Figure S3. Depletion of NOGO-A-positive mature oligodendrocytes during demyelination in the midline of the corpus callosum of aged mice, Figure S4. Accumulation of microglia during demyelination in the medial corpus callosum of aged mice, Figure S5. Changes of different glia populations during demyelination in the lateral parts of the corpus callosum in aged mice, Figure S6. MBP re-expression during remyelination in the medial corpus callosum of aged mice, Figure S7. Repopulation of mature oligodendrocytes during remyelination in the central corpus callosum of aged mice, Figure S8. Decrease of microglia during the process of remyelination in the medial part of the corpus callosum of aged mice, Figure S9. Changes of myelin and glia cell populations during remyelination in the lateral parts of the corpus callosum in aged mice, Figure S10. Correlation of myelin changes and microglia reaction during de- and remyelination in aged mice.
