# Supplementary material for: Delayed Demyelination and Impaired Remyelination in Aged Mice in the Cuprizone Model
Source: Cells. 2020 Apr 11;9(4):945. doi: 10.3390/cells9040945 (PMC7226973; doi:10.3390/cells9040945)
Supplement: Supplementary file 1 [file cells-09-00945-s001.zip › Supplemental figures.pdf]

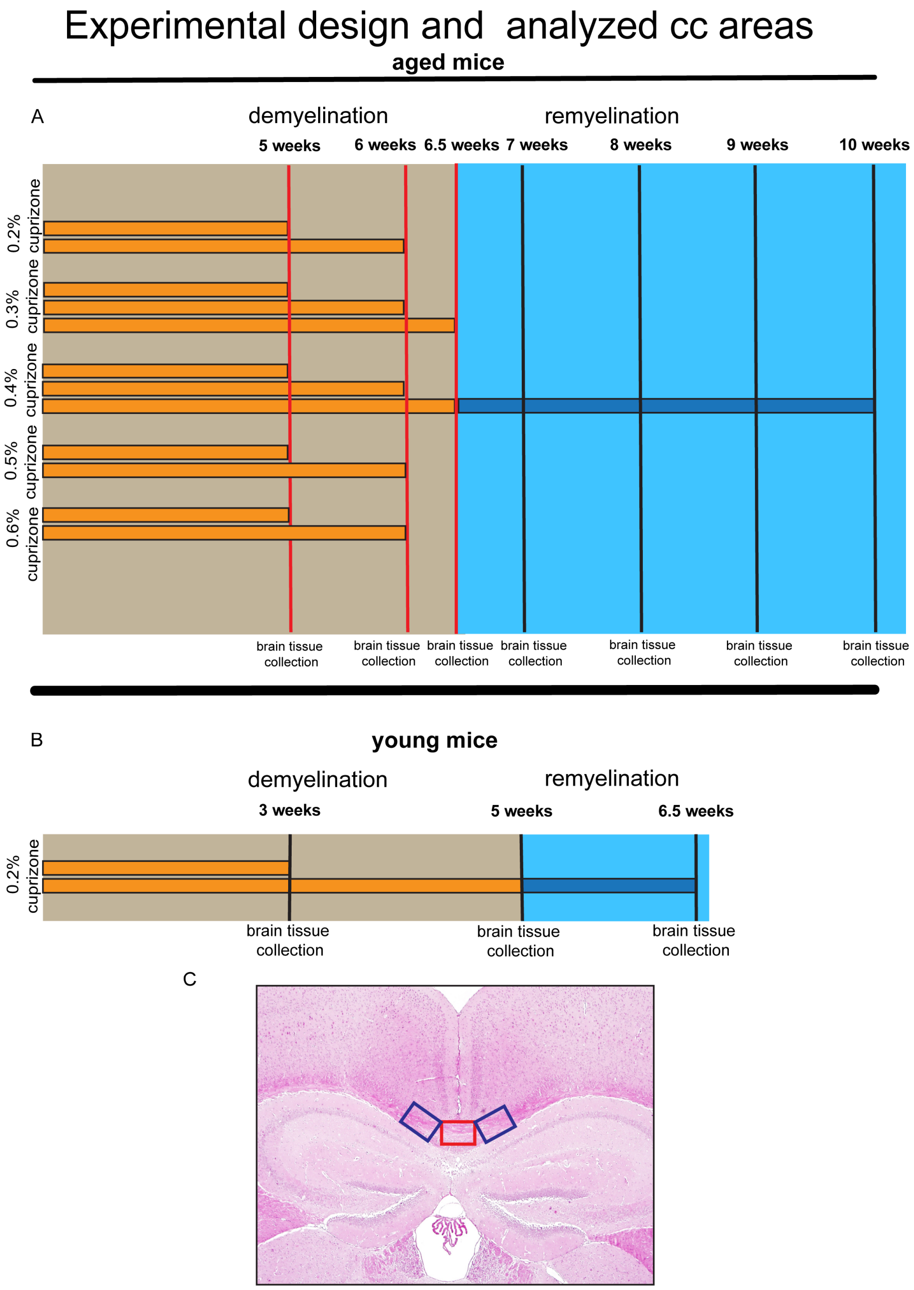


**Supplemental Figure 1**

Supplemental Figure 1. Experimental design.

At first, 6 months old aged mice were fed with different concentrations of cuprizone (0.2% - 0.6%) mixed into ground standard rodent chow for different periods of time (5 or 6 weeks) (A). Secondly, treatment with 0.4% cuprizone was extended for another half week to induce complete demyelination in the corpus callosum of aged mice (6.5 weeks). Subsequently, aged mice received standard rodent chow in order to evaluate the course of remyelination at different time points (7, 8, 9, 10 weeks). 2 months old young mice were fed with 0.2% cuprizone mixed into milled standard rodent chow for 5 weeks in order to induce complete demyelination in the corpus callosum. An additional group (B) of young mice received 0.2% cuprizone for 3 weeks. Remyelination was evaluated 1.5 weeks after cessation of cuprizone feeding at 6.5 weeks. Each group consisted of 5-6 animals. (C) H&E (hematoxylin-eosin)-stained control mouse brain. The boxed areas represent the areas of the corpus callosum that were analyzed (red: medial corpus callosum, blue: lateral corpus callosum).

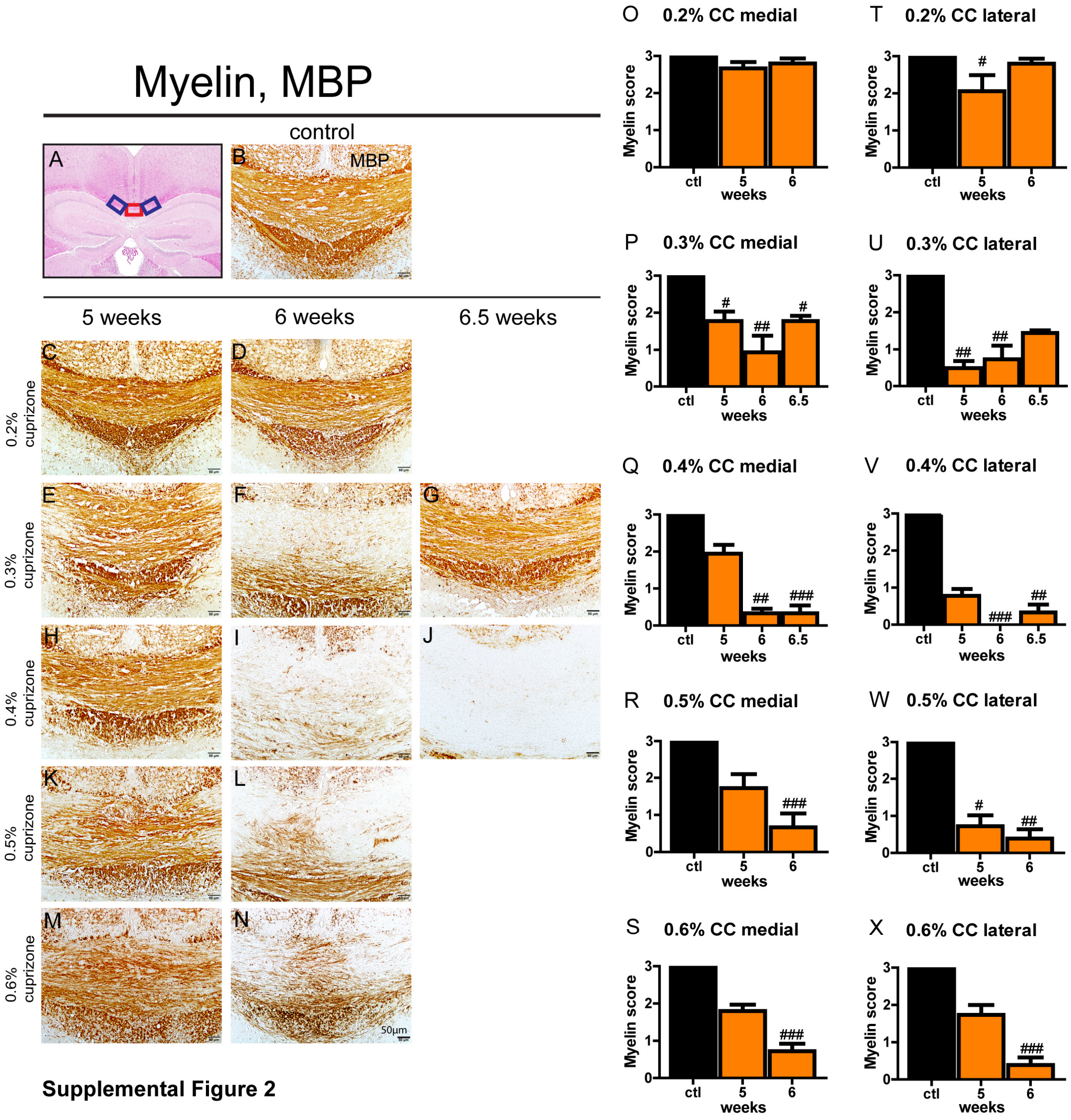


**Supplemental Figure 2**

Supplemental Figure 2. MBP loss in the corpus callosum of aged mice after different cuprizone treatment protocols.

The H&E-stained control mouse brain (A) contains the areas of the corpus callosum that were analyzed (red box: medial part; blue boxes: lateral parts). Representative MBP-stained sections (C-N) demonstrate reduction of MBP staining during demyelination in the central corpus callosum of aged mice after cuprizone treatment with different doses (0.2% - 0.6% cuprizone) for different feeding periods (5, 6 or 6.5 weeks). A representative picture (B) shows MBP staining in the midline of the corpus callosum of an age-matched control animal. A myelination score of 3 represents complete myelination, whereas a score of 0 represents complete demyelination. Graphs display the myelin score per time point and group in the midline (O-S) and in the lateral part of the corpus callosum (T-X) in aged animals. Bars represent mean + SEM. Significant effects between different investigated time points are indicated by asterisks and effects in comparison to control are indicated by hashmarks (*/# $p<0.05$; **/## $p<0.01$; ***/### $p<0.001$). Ctl = control; 5, 6 or 6.5 weeks = feeding period of cuprizone for respective duration. N = 5-6 animals per group.

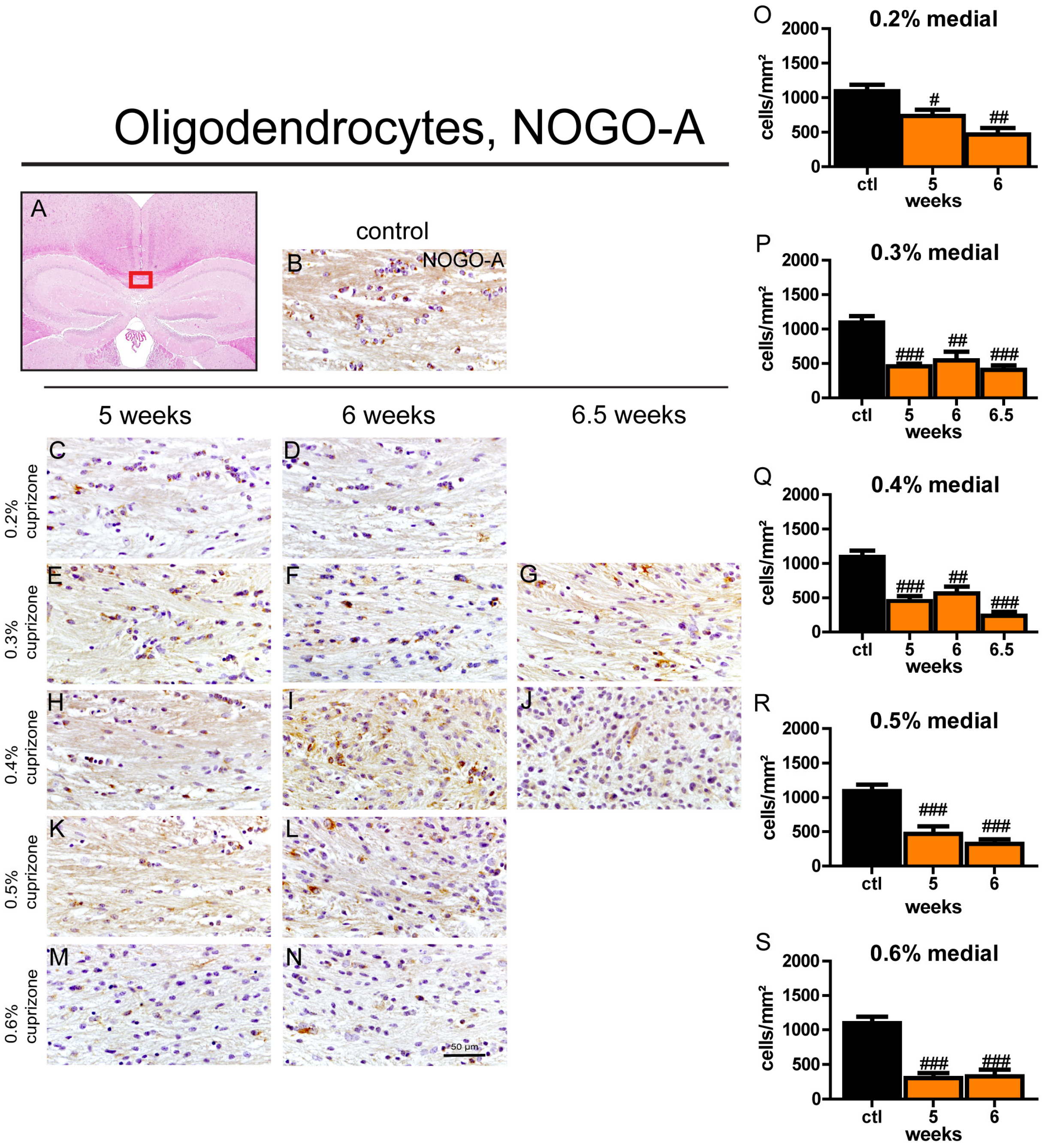


**Supplemental Figure 3**

Supplemental Figure 3. Depletion of NOGO-A-positive mature oligodendrocytes during demyelination in the midline of the corpus callosum of aged mice.

The outlined area (red box) shows the medial part of the corpus callosum which was investigated (A). Representative NOGO-A–stained sections (C-N) show the depletion of oligodendrocytes during demyelination in the midline of the corpus callosum of aged mice after cuprizone treatment with different concentrations (0.2% - 0.6% cuprizone) for different time periods (5, 6 or 6.5 weeks). An exemplary brain section of a control animal is shown (B). Graphs (O-S) represent numbers of NOGO-A-positive cells/mm² in the medial part of the corpus callosum in aged animals. Bars display mean + SEM. Significant effects between different investigated time points are indicated by asterisks and effects in comparison to control are indicated by hashmarks (*/# $p<0.05$; **/## $p<0.01$; ***/### $p<0.001$). Ctl = control; 5, 6 or 6.5 weeks = feeding period of cuprizone for respective duration. n = 5-6 animals per group.

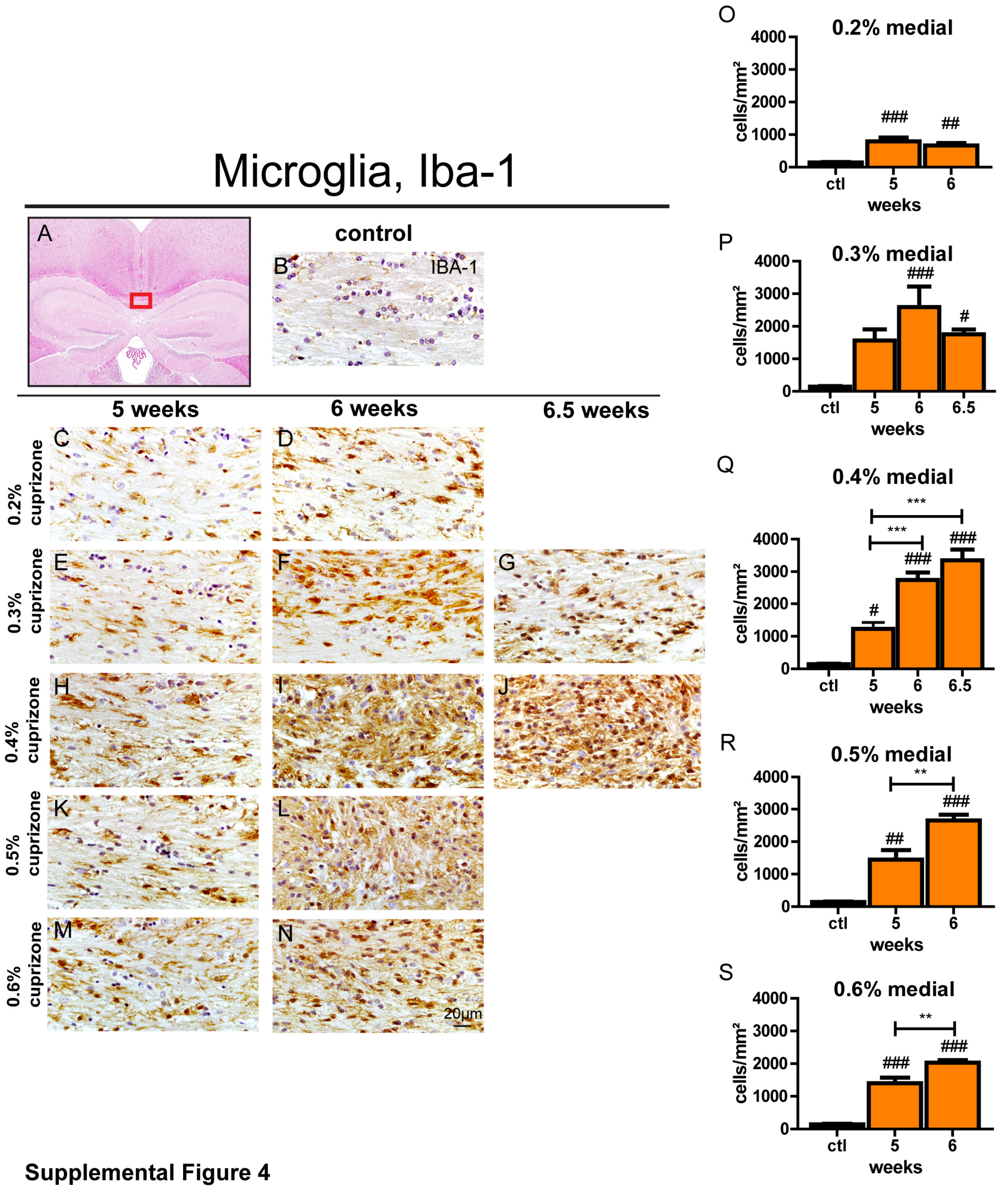


**Supplemental Figure 4**

Supplemental Figure 4. Accumulation of microglia during demyelination in the medial corpus callosum of aged mice.

The red box in (A) displays the medial section of the corpus callosum which was analyzed. Exemplary images (C-N) of Iba-1-stained brain sections show microglia accumulation during demyelination in the medial part of the corpus callosum of aged mice after cuprizone treatment with various doses (0.2% - 0.6% cuprizone) for different feeding periods (5, 6 or 6.5 weeks). A representative brain section of a control animal stained for Iba-1 is shown in B. Graphs depict the amount of Iba-1 positive cells/mm$^2$ per time point in the midline of corpus callosum in aged animals (O-S). Bars represent mean + SEM. Effects between different investigated time points are indicated by asterisks and effects in comparison to control are indicated by hash marks (*/# p<0.05; **/## p<0.01; ***/### p<0.001). Ctl = control; 5, 6 or 6.5 weeks weeks = feeding period of cuprizone for respective duration. N = 5-6 animals per group.

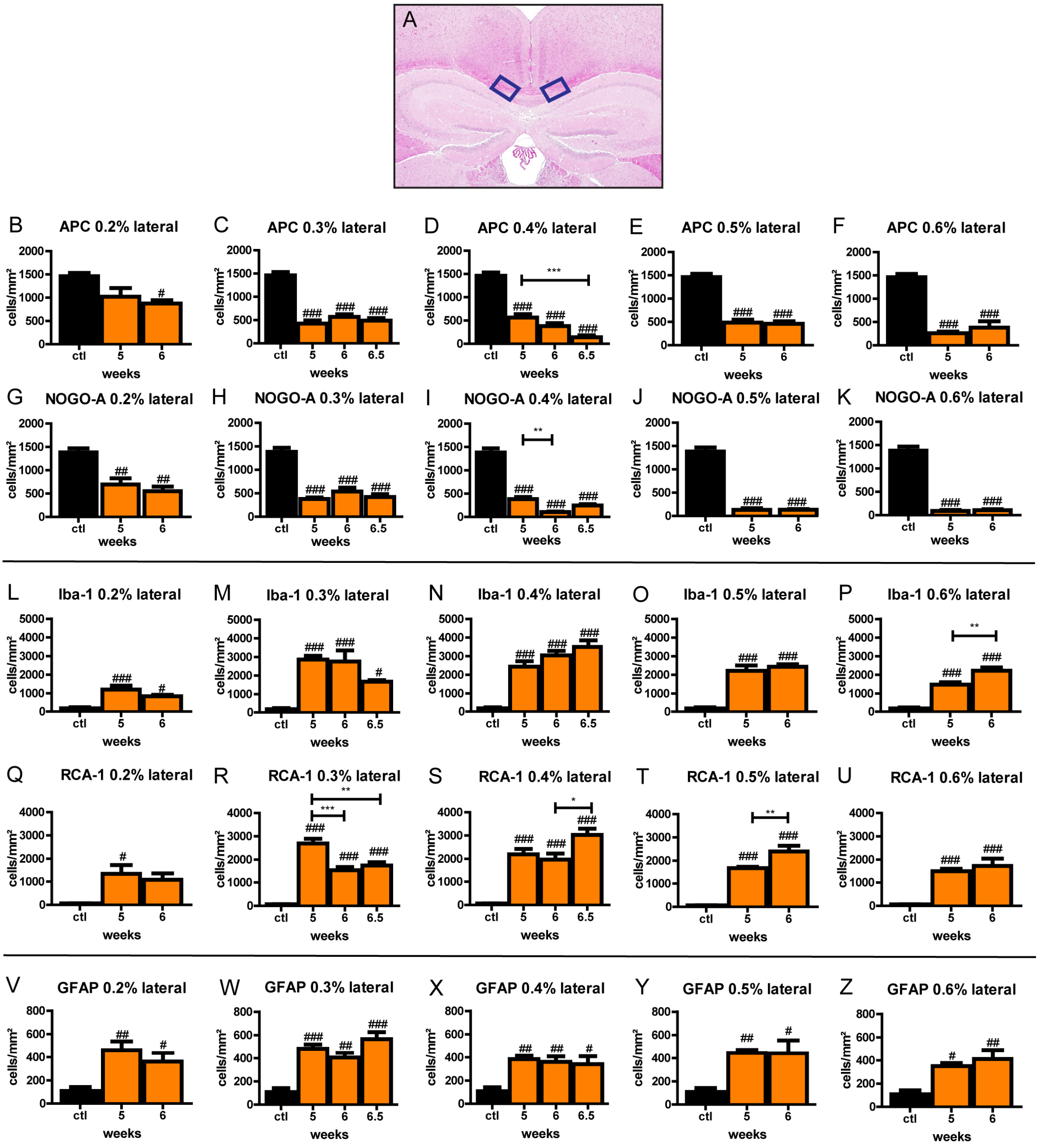


## Supplemental Figure 5

Supplemental Figure 5. Changes of different glia populations during demyelination in the lateral parts of the corpus callosum in aged mice.

The marked areas (blue boxes) in A outline the lateral parts of the corpus callosum that were analyzed. Graphs represent numbers of different glial cells (number of cells/mm$^2$ at the respective time point) in the lateral parts of the corpus callosum in aged animals during cuprizone feeding with different concentrations (0.2% - 0.6% cuprizone) for different treatment periods (5, 6 or 6.5 weeks). Depletion of mature oligodendrocytes is demonstrated by staining for APC (B-F) and NOGO-A (G-K). Microglia accumulation and activation is shown by Iba-1 (L-P) and RCA-1 (Q-U) stainings. Astrocytosis during demyelination is visualized by GFAP (V-Z). Bars represent mean + SEM. Significant effects between different investigated time points are indicated by asterisks and effects in comparison to control are indicated by hashmarks (*/# $p<0.05$; **/## $p<0.01$; ***/### $p<0.001$). Ctl = control; 5, 6 or 6.5 weeks = treatment period with cuprizone for respective duration. N = 5-6 animals per group.

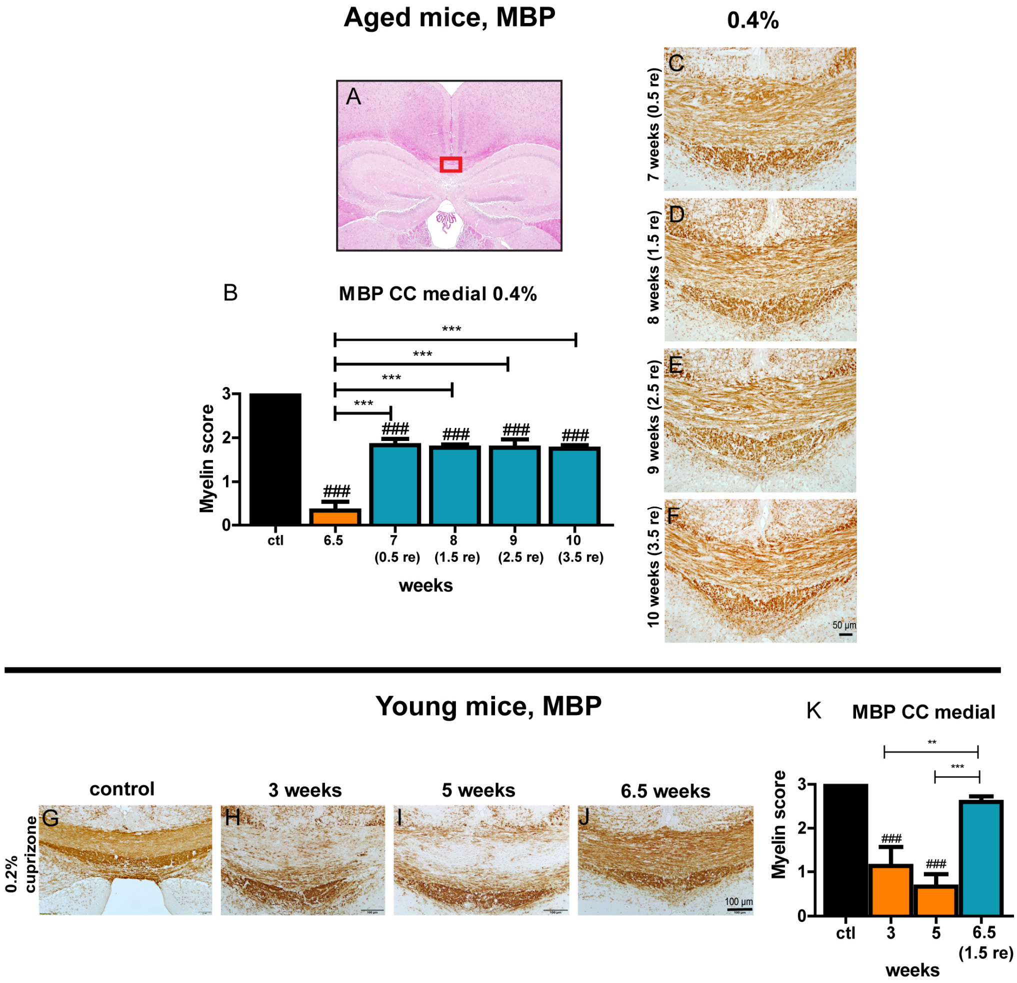


**Supplemental Figure 6**

Supplemental Figure 6. MBP re-expression during remyelination in the medial corpus callosum of aged mice.

MBP re-expression during remyelination in the midline of the corpus callosum of aged mice. The area of the corpus callosum that was analyzed is highlighted (A). B shows the myelin score for MBP in the midline of corpus callosum in aged animals fed for 6.5 weeks with 0.4% cuprizone and subsequent remyelination (0.5, 1.5, 2.5, and 3.5 weeks after cessation of treatment with cuprizone). A score of 3 represents complete myelination, whereas a score of 0 represents complete demyelination. Representative MBP-stained sections (C-F) display the course of remyelination in aged mice. See Supplemental Figure 2 for a representative image of aged mice for control and demyelination after 0.4% cuprizone feeding for 6.5 weeks. The myelin score for young mice treated with 0.2% cuprizone for 5 weeks and subsequent remyelination for 1.5 weeks is shown in K. Representative MBP-stained sections show the course of de- and remyelination in young mice (G-J). Bars display mean + SEM. Significant effects between different investigated time points are indicated by asterisks and effects in comparison to control are indicated by hashmarks (*/# $p<0.05$; **/## $p<0.01$; ***/### $p<0.001$). Ctl = control; 6.5 weeks= feeding period of 0.4% cuprizone in aged mice; 7 (0.5 re) weeks = 0.5 weeks of remyelination, 8 (1.5 re) weeks = 1.5 weeks of remyelination, 9 (2.5 re) weeks = 2.5 weeks of remyelination, 10 (3.5 re) weeks = 3.5 weeks of remyelination in aged mice. 3 and 5 weeks = feeding period of 0.2% cuprizone in young mice; 6.5 (1.5) weeks = 1.5 weeks of remyelination in young mice. N = 5-6 animals per group.

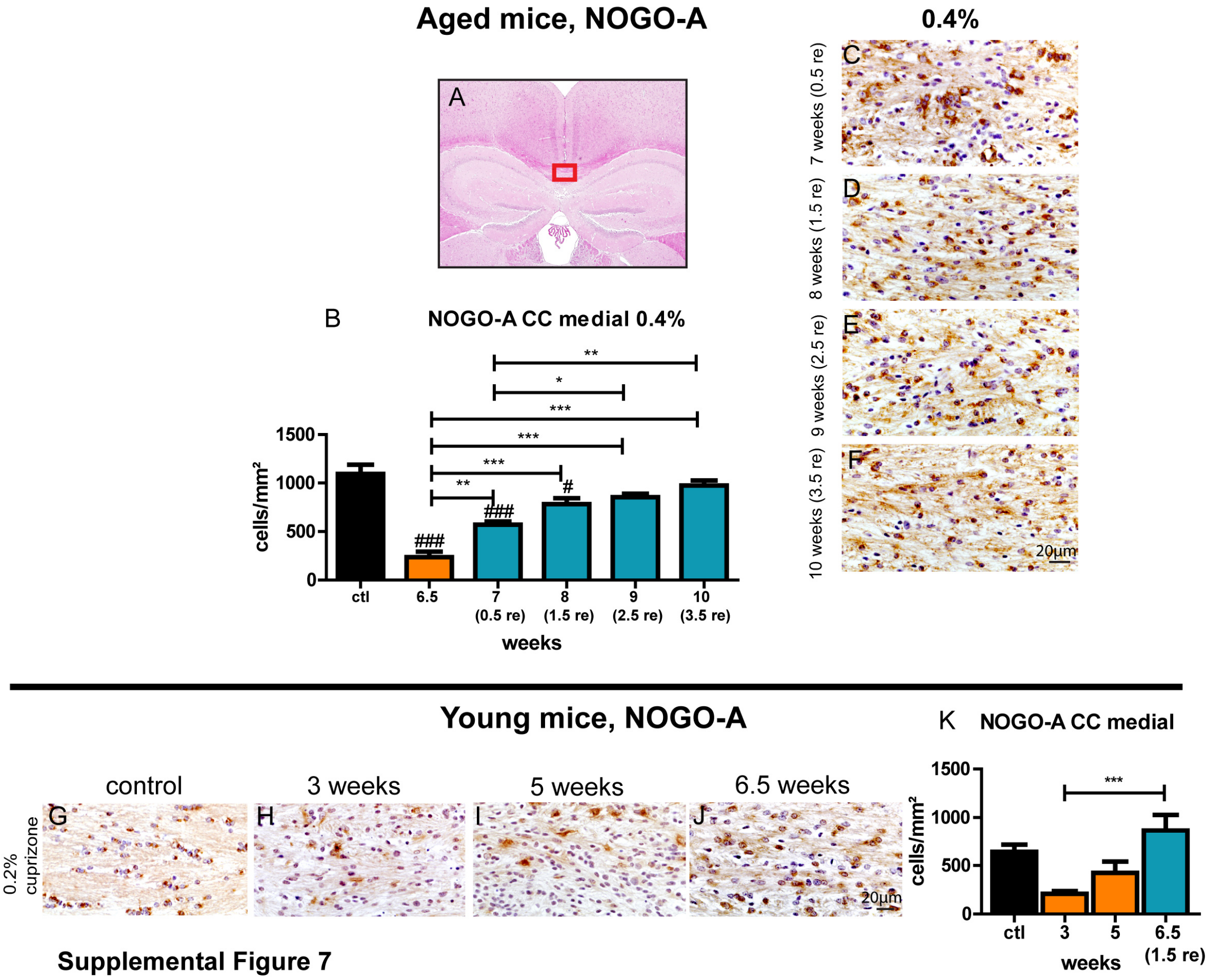


Supplemental Figure 7. Repopulation of mature oligodendrocytes during remyelination in the central corpus callosum of aged mice.

The red box in (A) displays the medial section of the corpus callosum which was analyzed. Graphs show the number of NOGO-A-positive oligodendrocytes/mm² for the respective time points of demyelination and subsequent remyelination in aged mice fed for 6.5 weeks with 0.4% cuprizone (B) and young mice treated for 5 weeks with 0.2% cuprizone (K). See Supplemental Figure 3 for representative sections of control and demyelination after 0.4% cuprizone feeding for 6.5 weeks, respectively. Exemplary images show repopulation of NOGO-A-positive oligodendrocytes during the process of remyelination in the medial corpus callosum of aged mice (C-F) and during de- and remyelination in young mice (G-J). Bars show mean + SEM. Significant effects between different investigated time points are indicated by asterisks and effects in comparison to control are indicated by hashmarks (*/# $p<0.05$; **/## $p<0.01$; ***/### $p<0.001$). Ctl = control; 6.5 weeks= feeding period of 0.4% cuprizone in aged mice; 7 (0.5 re) weeks = 0.5 weeks of remyelination, 8 (1.5 re) weeks = 1.5 weeks of remyelination, 9 (2.5 re) weeks = 2.5 weeks of remyelination, 10 (3.5 re) weeks = 3.5 weeks of remyelination in aged mice. 3 and 5 weeks = feeding period of 0.2% cuprizone in young mice; 6.5 (1.5) weeks = 1.5 weeks of remyelination in young mice. N = 5-6 animals per group.

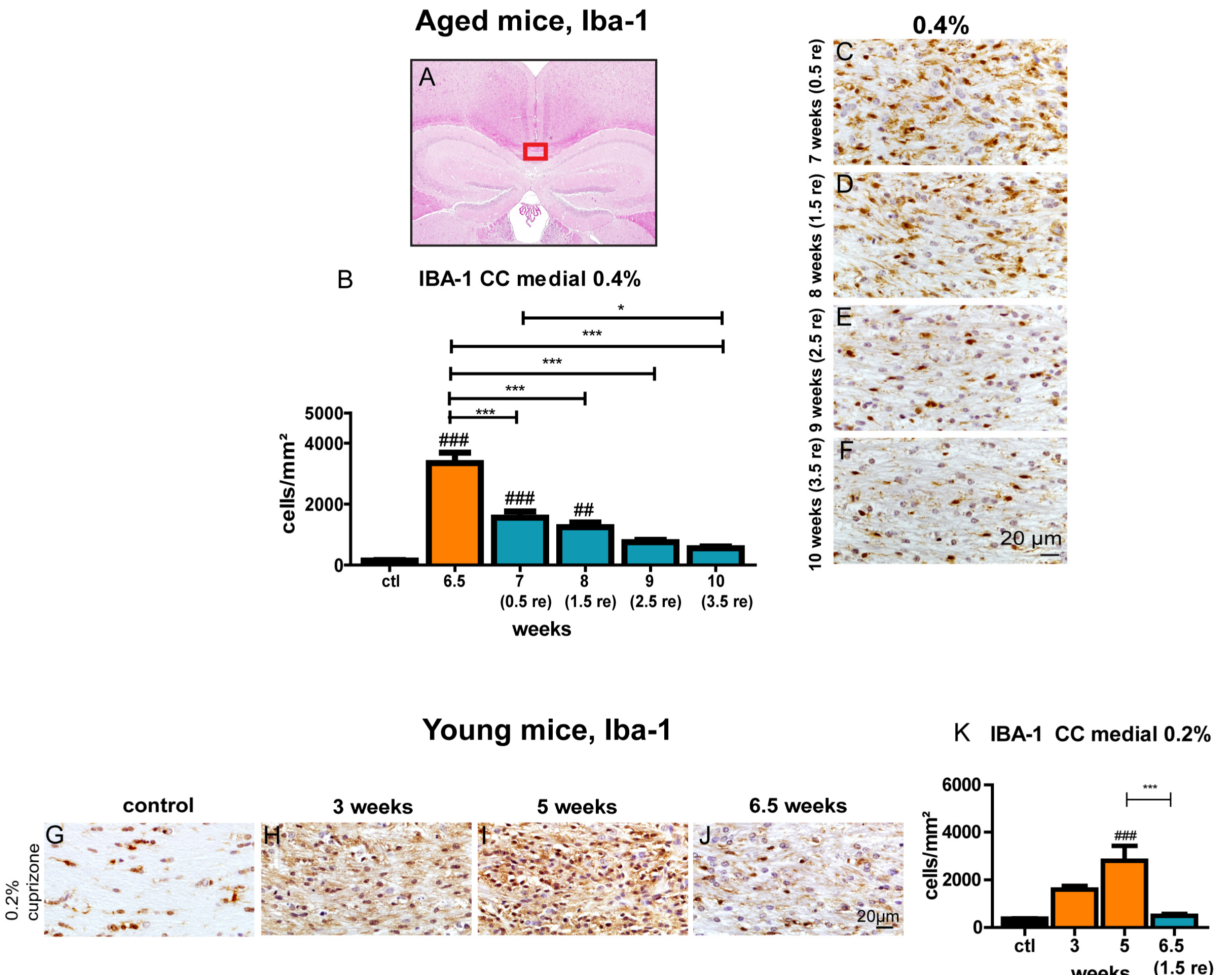


**Supplemental Figure 8**

Supplemental Figure 8. Decrease of microglia during the process of remyelination in the medial part of the corpus callosum of aged mice.

The medial part of the corpus callosum which was analyzed is highlighted (A). Graphs represent number of cells/mm$^2$ of IBA-1-positive microglia per time point and group in aged animals (B) fed for 6.5 weeks with 0.4% cuprizone and young mice treated with cuprizone for 5 weeks with 0.2% (K). Representative images show the decline of microglia accumulation during remyelination in the central corpus callosum of aged mice (C-F) and the increase of microglia during demyelination and subsequent decrease during remyelination in young mice (G-J). For exemplary sections of aged controls and of demyelination after 0.4% cuprizone feeding for 6.5 weeks, see Supplemental Figure 4. Bars show mean + SEM. Significant effects between different analyzed time points are indicated by asterisks and effects in comparison to control are indicated by hashmarks (*/# $p<0.05$; **/## $p<0.01$; ***/### $p<0.001$). Ctl = control; 6.5 weeks = feeding period of 0.4% cuprizone in aged mice; 7 (0.5 re) weeks = 0.5 weeks of remyelination, 8 (1.5 re) weeks = 1.5 weeks of remyelination, 9 (2.5 re) weeks = 2.5 weeks of remyelination, 10 (3.5 re) weeks = 3.5 weeks of remyelination in aged mice. 3 and 5 weeks = feeding period of 0.2% cuprizone in young mice; 6.5 (1.5) weeks = 1.5 weeks of remyelination in young mice. N = 5-6 animals per group.

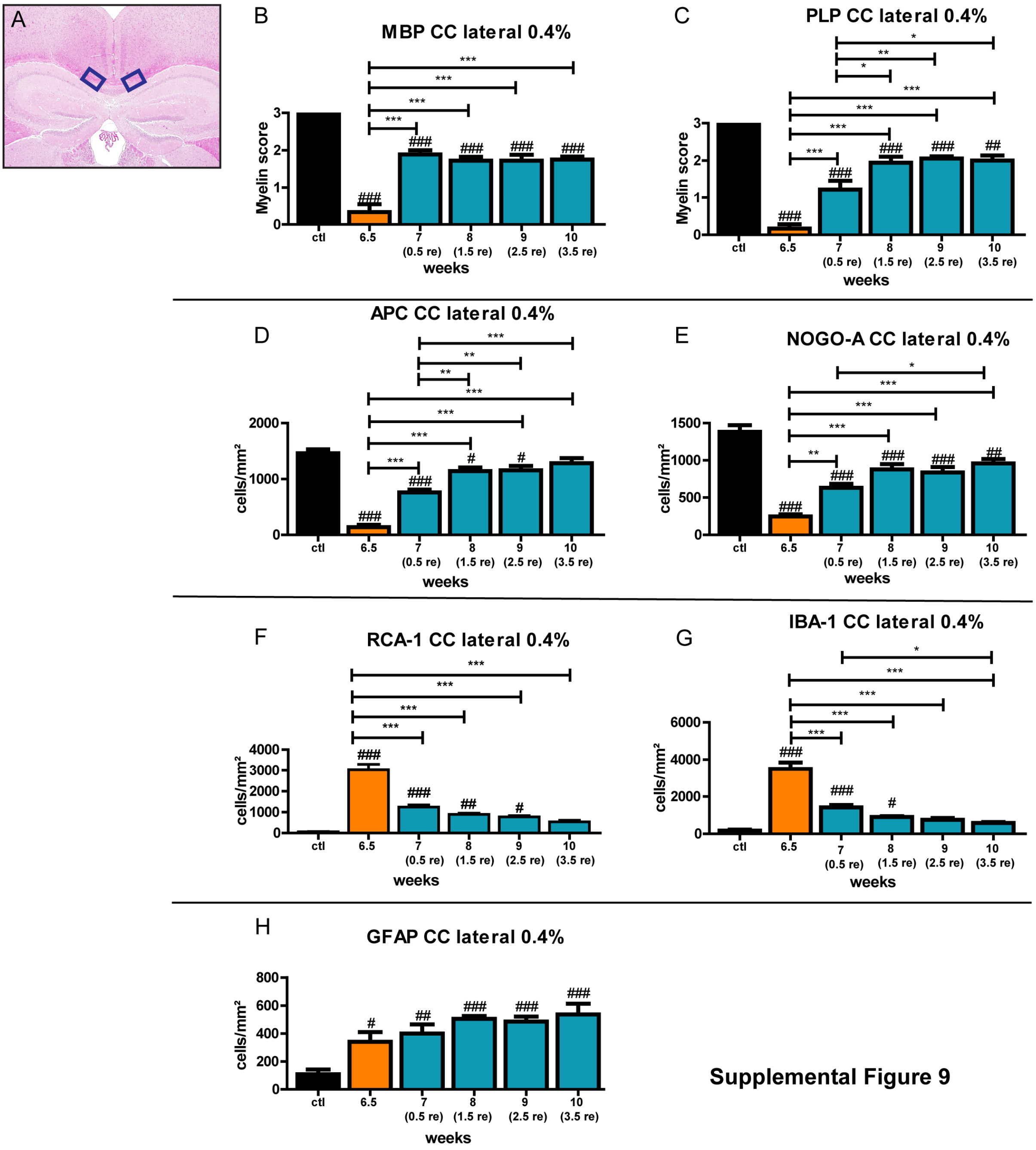


Supplemental Figure 9. Changes of myelin and glia cell populations during remyelination in the lateral parts of corpus callosum in aged mice.

The boxed areas in A outline the lateral parts of the corpus callosum that were examined. After demyelination with 0.4% cuprizone for 6.5 weeks the lateral segments of the corpus callosum demonstrate re-expression of myelin proteins MBP (B) and PLP (C), repopulation of APC-positive- and NOGO-A-positive oligodendrocytes (D, E), reduction of microglia (F, G) and persisting astrocytosis (H) during the remyelination period. Graphs show myelination score for myelin proteins (a score of 3 represents complete myelination, whereas a score of 0 represents complete demyelination) and absolute cell glia cells numbers/mm² for the respective time points of de- and remyelination. Bars show mean + SEM. Significant effects between different analyzed time points are indicated by asterisks and effects in comparison to control are indicated by hashmarks (*/# $p<0.05$; **/## $p<0.01$; ***/### $p<0.001$). Ctl = control; 6.5 weeks = feeding period of 0.4% cuprizone in aged mice; 7 (0.5 re) weeks = 0.5 weeks of remyelination, 8 (1.5 re) weeks = 1.5 weeks of remyelination, 9 (2.5 re) weeks = 2.5 weeks of remyelination, 10 (3.5 re) weeks = 3.5 weeks of remyelination in aged mice. 3 and 5 weeks = feeding period of 0.2% cuprizone in young mice; 6.5 (1.5) weeks = 1.5 weeks of remyelination in young mice. N = 5-6 animals per group.

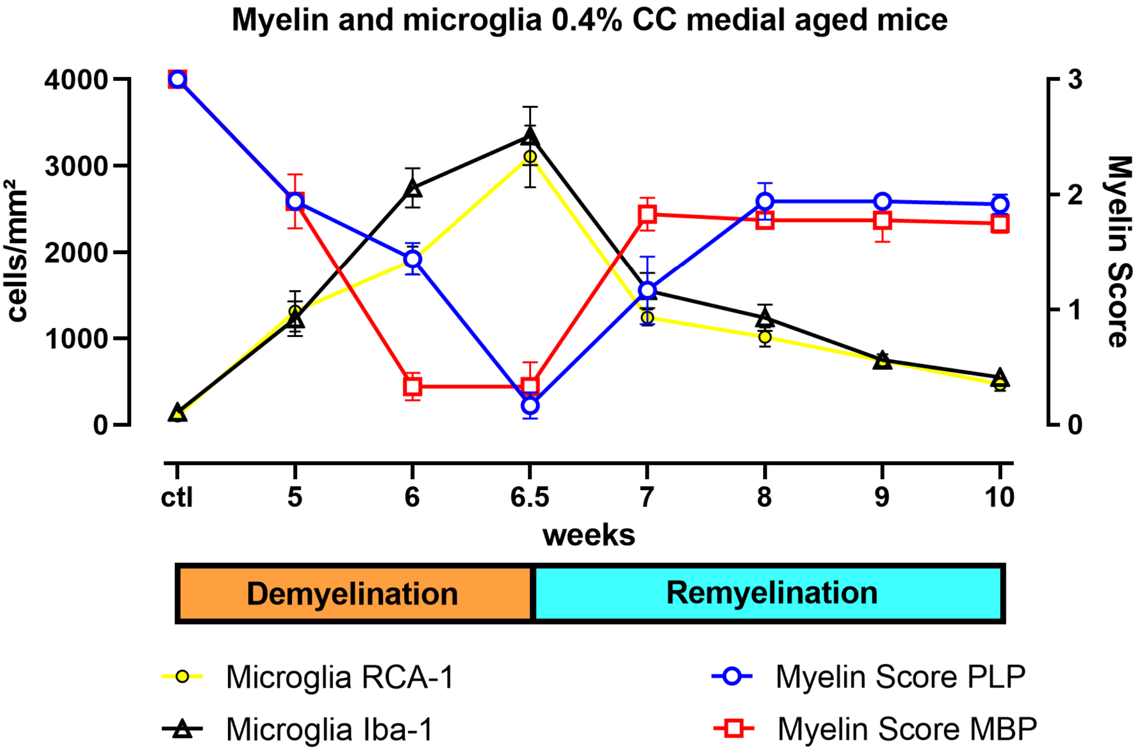


**Supplemental Figure 10**

Supplemental Figure 10. Correlation of myelin changes and microglia reaction during de- and remyelination in aged mice.

The graph visualizes cell numbers/mm² of RCA-1-positive activated microglia (yellow), Iba-1-positive microglia (black) with regard to the left y-axis and the myelin score for PLP (blue) and MBP (red) referring to the right y-axis. Single data points represent mean ± SEM.
