# Supplementary figures and images for: Delayed Demyelination and Impaired Remyelination in Aged Mice in the Cuprizone Model
Source: Cells. 2020 Apr 11;9(4):945. doi: 10.3390/cells9040945 (PMC7226973; doi:10.3390/cells9040945)

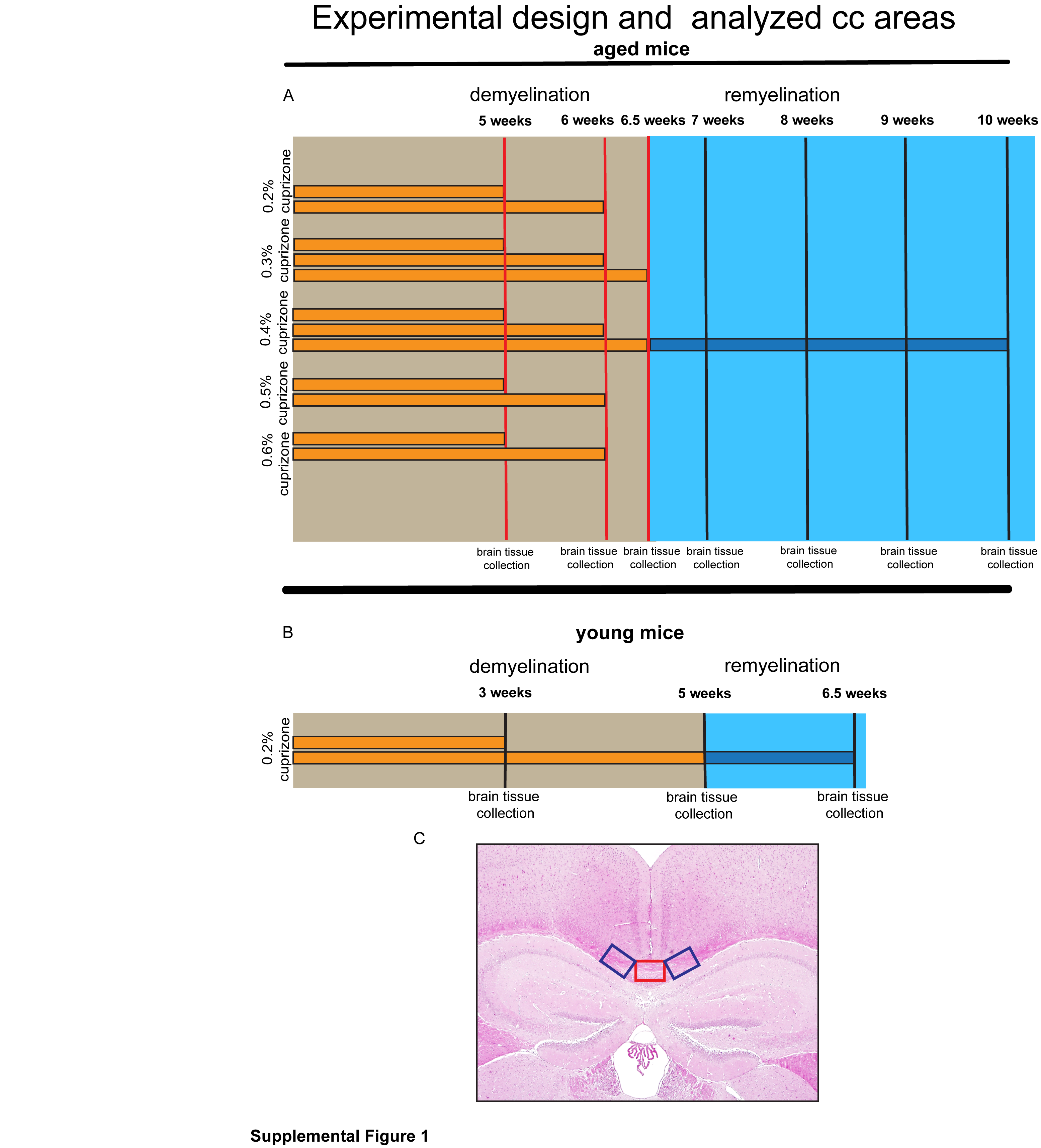

Supplement: Supplementary file 1 [file cells-09-00945-s001.zip › Supl 1_Experimental_Design.tif]

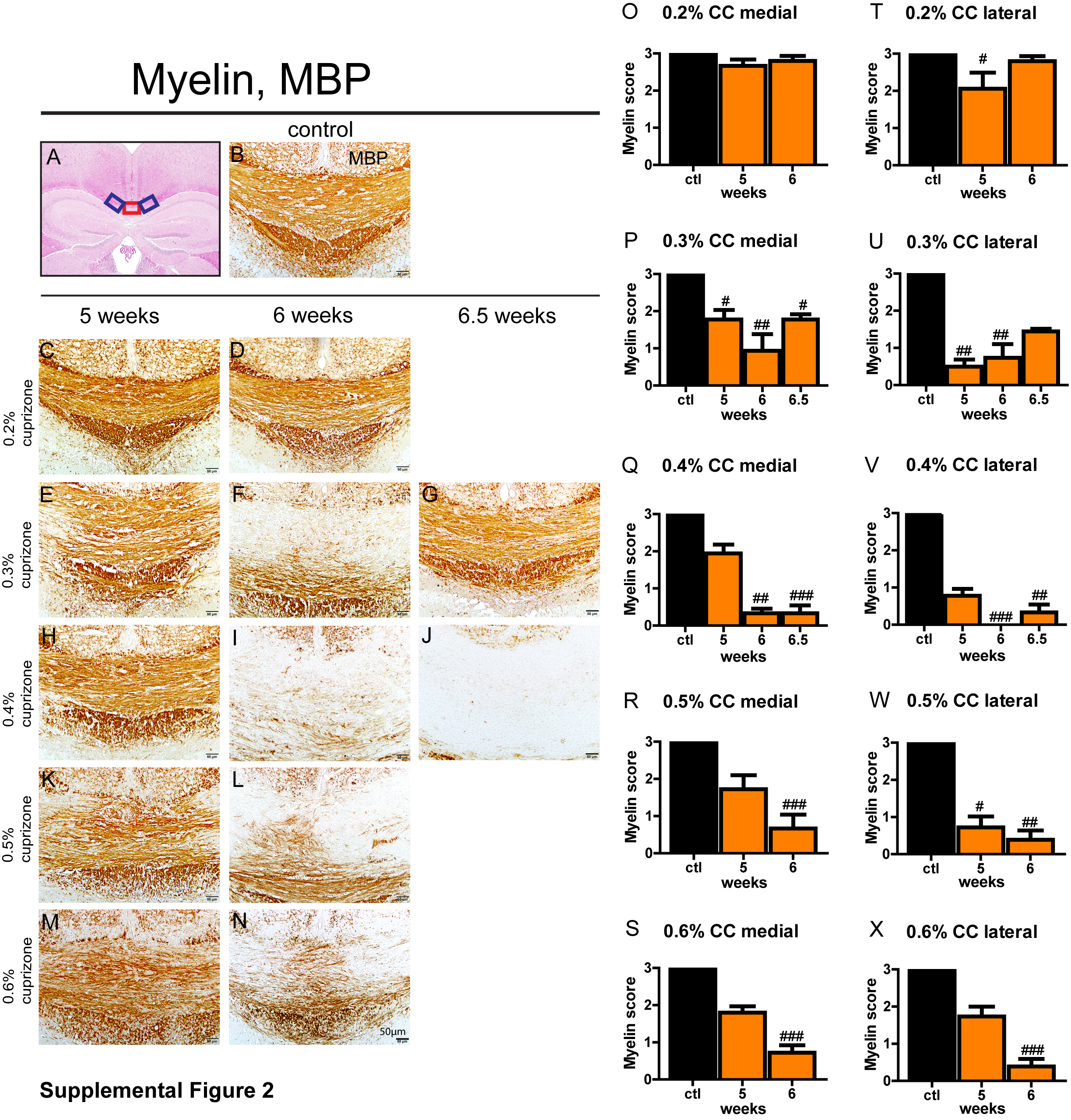

Supplement: Supplementary file 1 [file cells-09-00945-s001.zip › Supl 2_Myelin_MBP.tif]

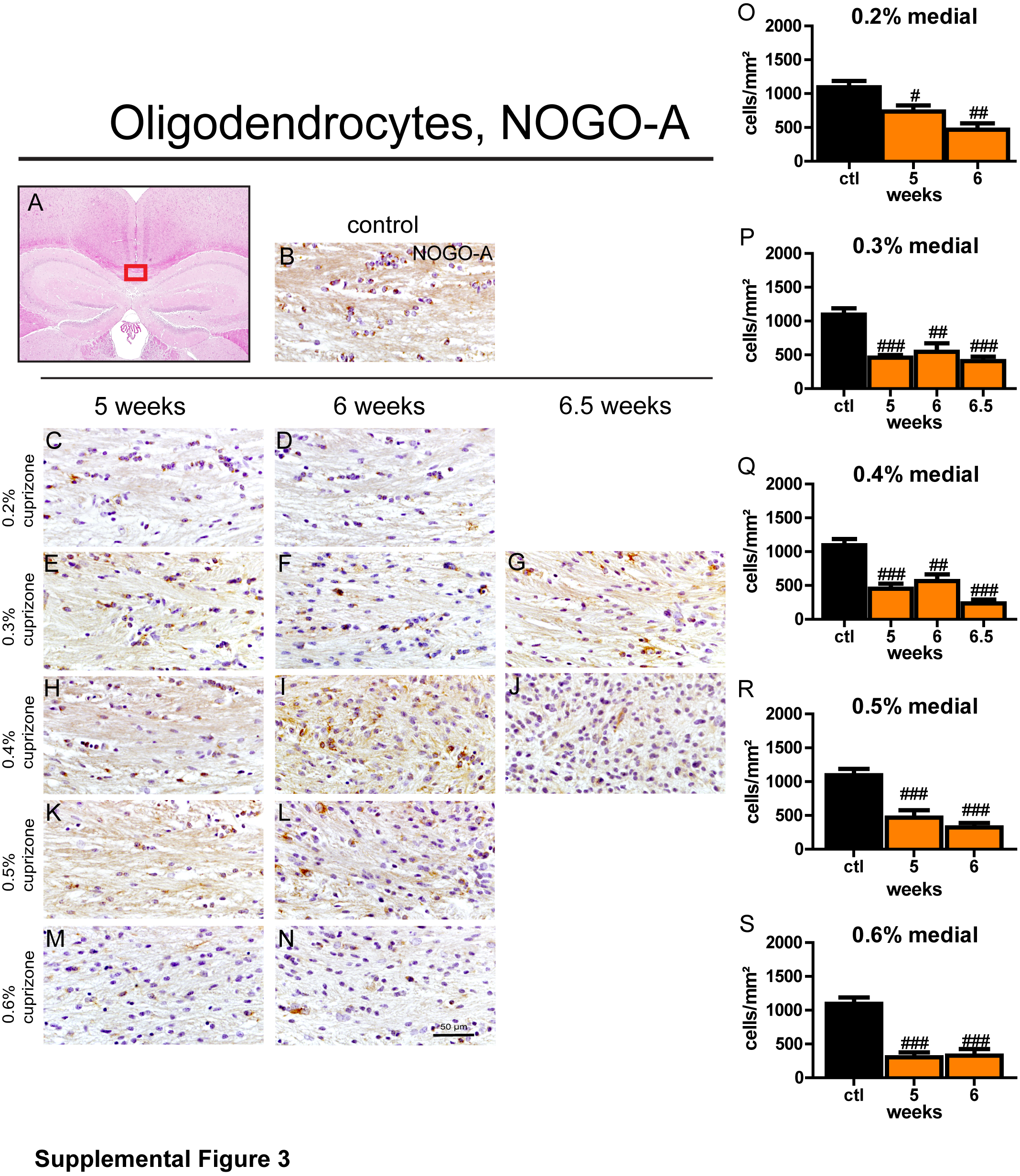

Supplement: Supplementary file 1 [file cells-09-00945-s001.zip › Supl 3_Oligodendrocytes_NOGO-A.tif]

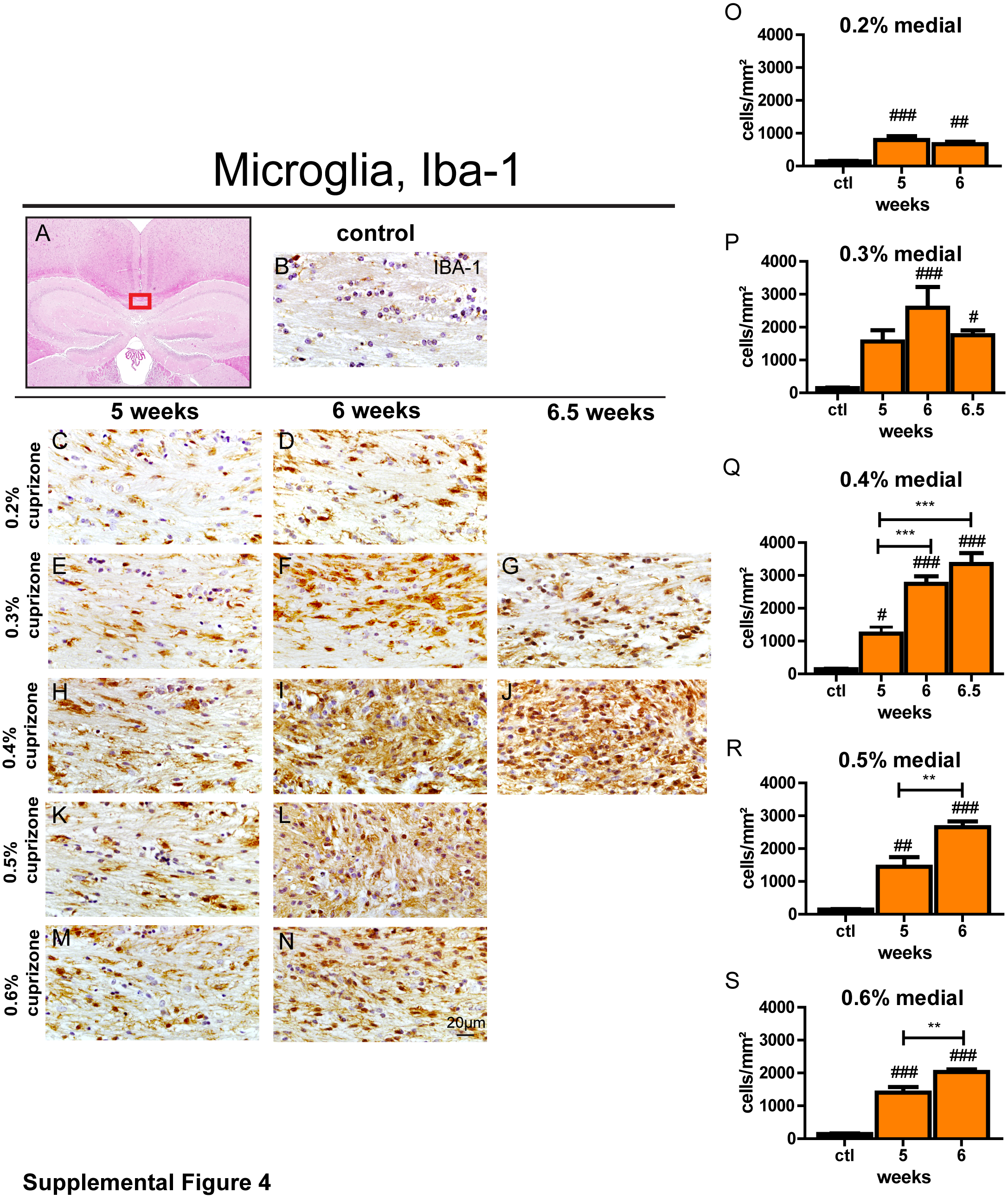

Supplement: Supplementary file 1 [file cells-09-00945-s001.zip › Supl 4_Microglia_Iba-1.tif]

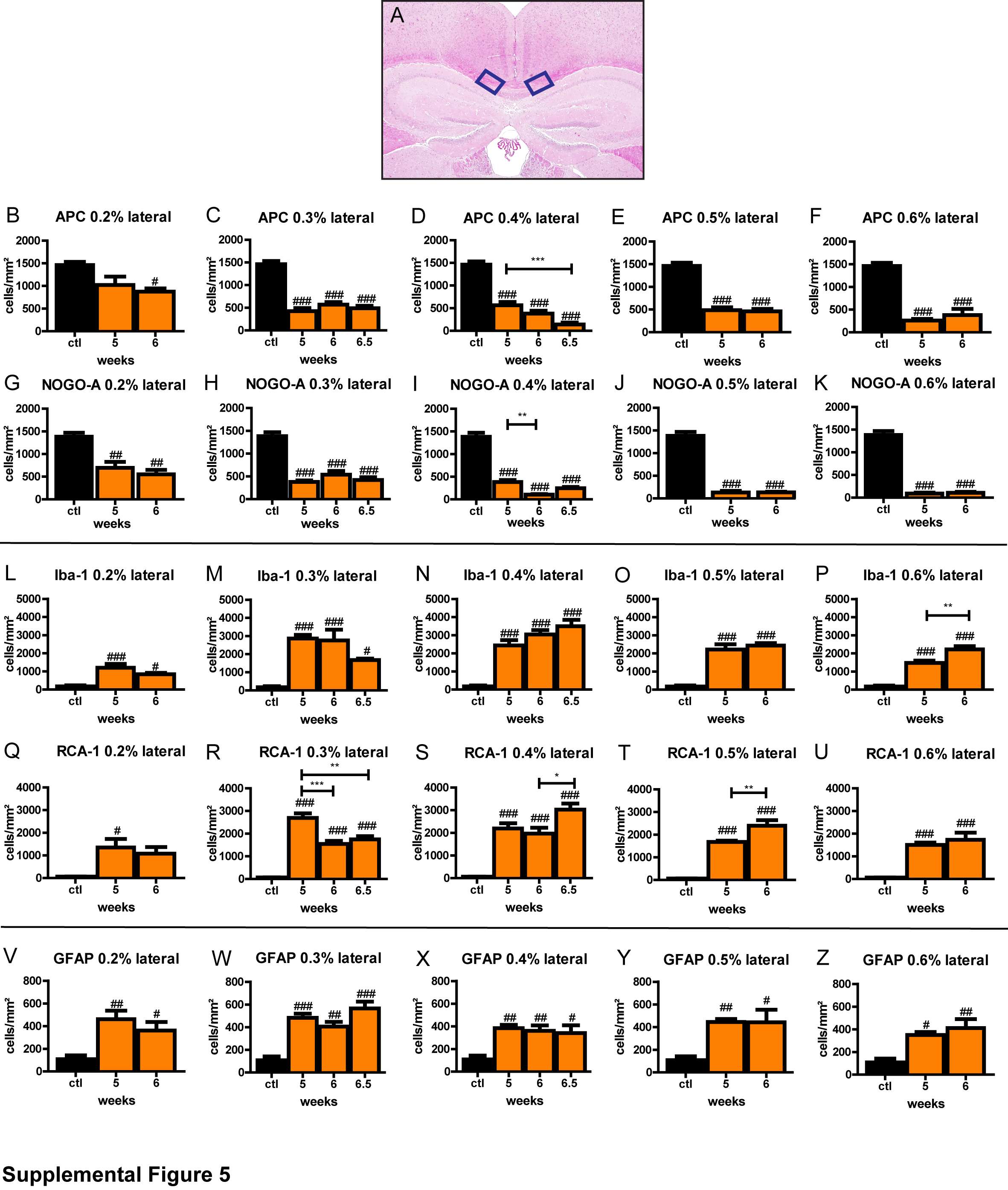

Supplement: Supplementary file 1 [file cells-09-00945-s001.zip › Supl 5_lateral cells.tif]

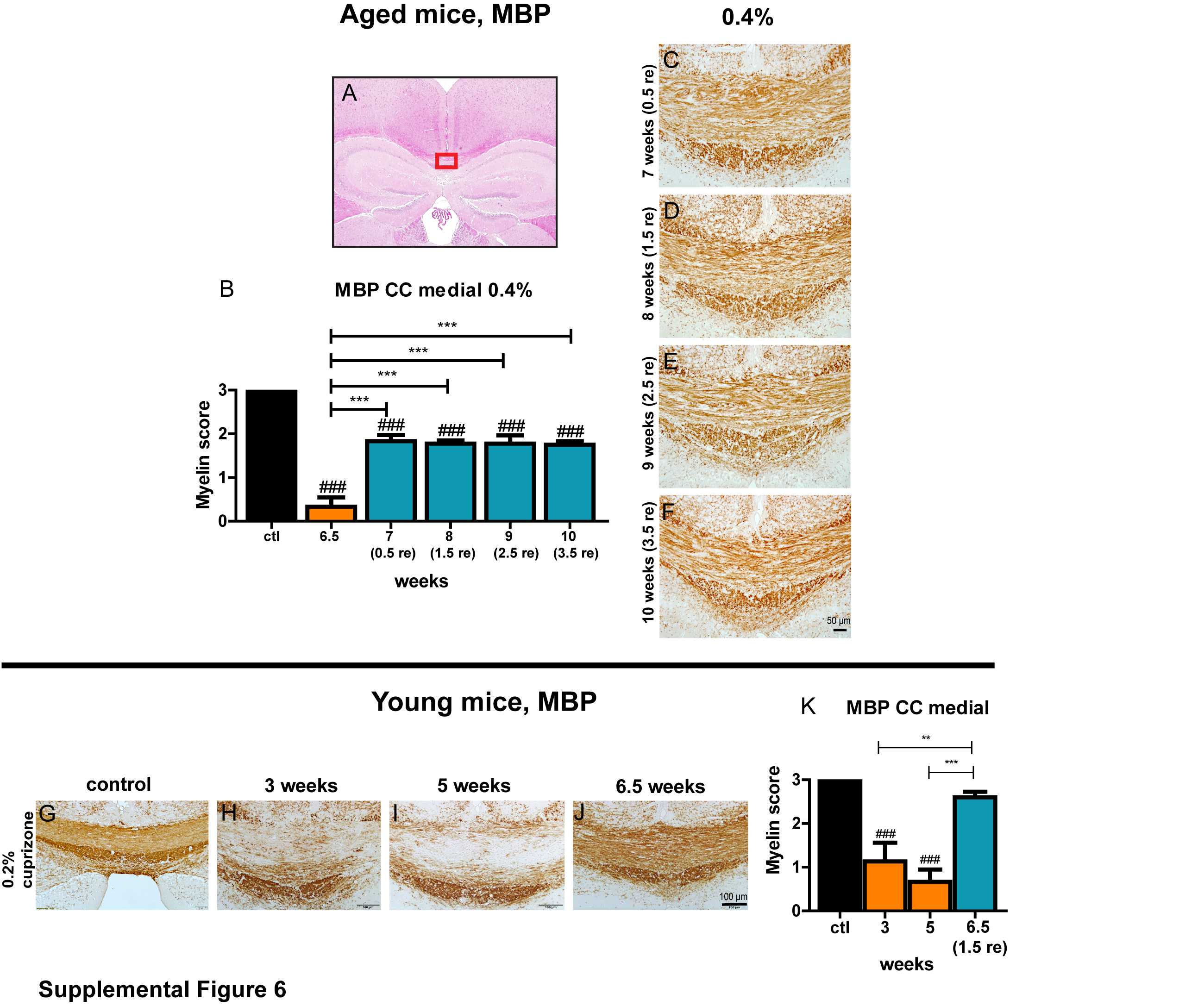

Supplement: Supplementary file 1 [file cells-09-00945-s001.zip › Supl 6_Myelin_MBP.tif]

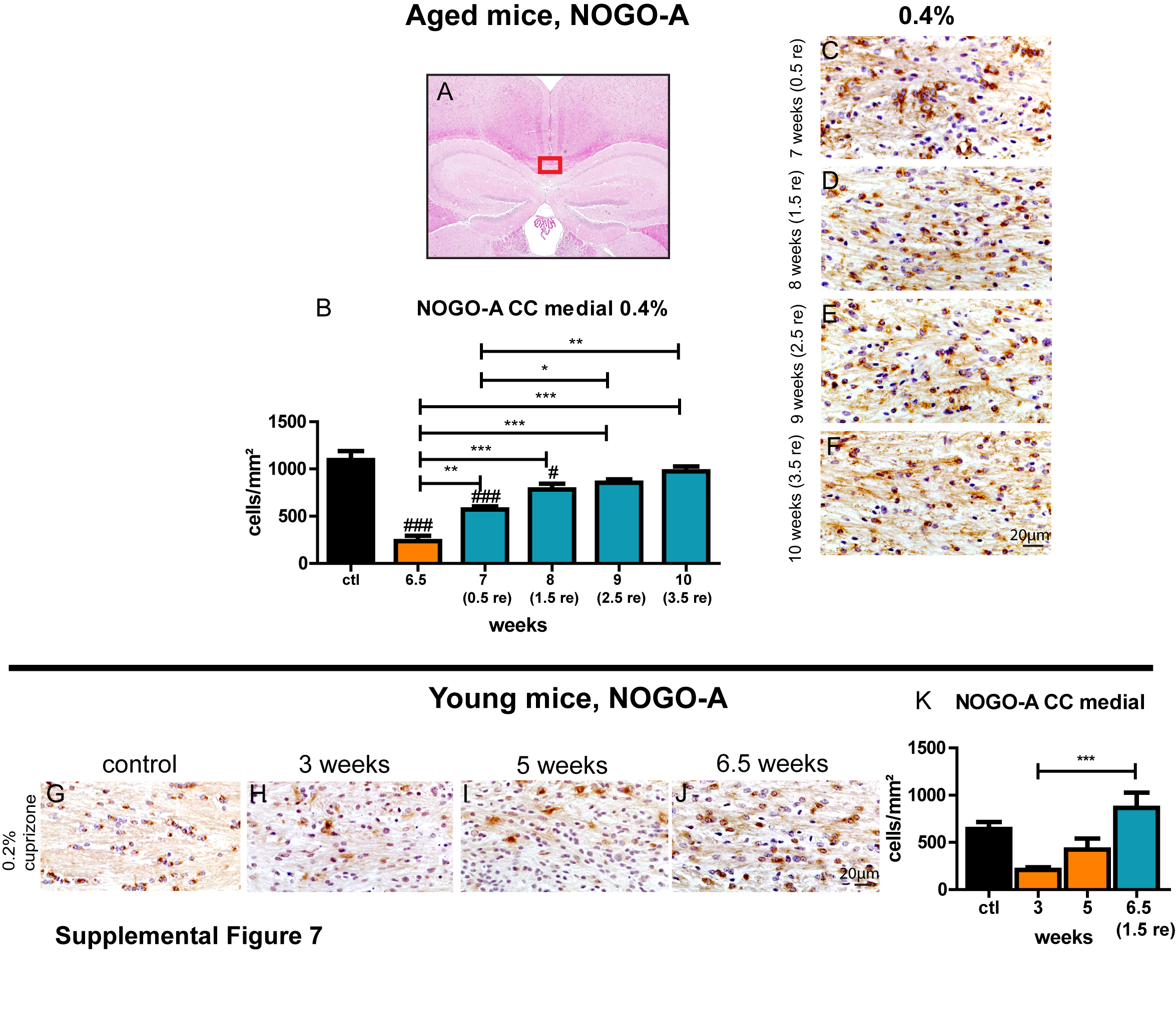

Supplement: Supplementary file 1 [file cells-09-00945-s001.zip › Supl 7_Oligodendrocytes_NOGO-A.tif]

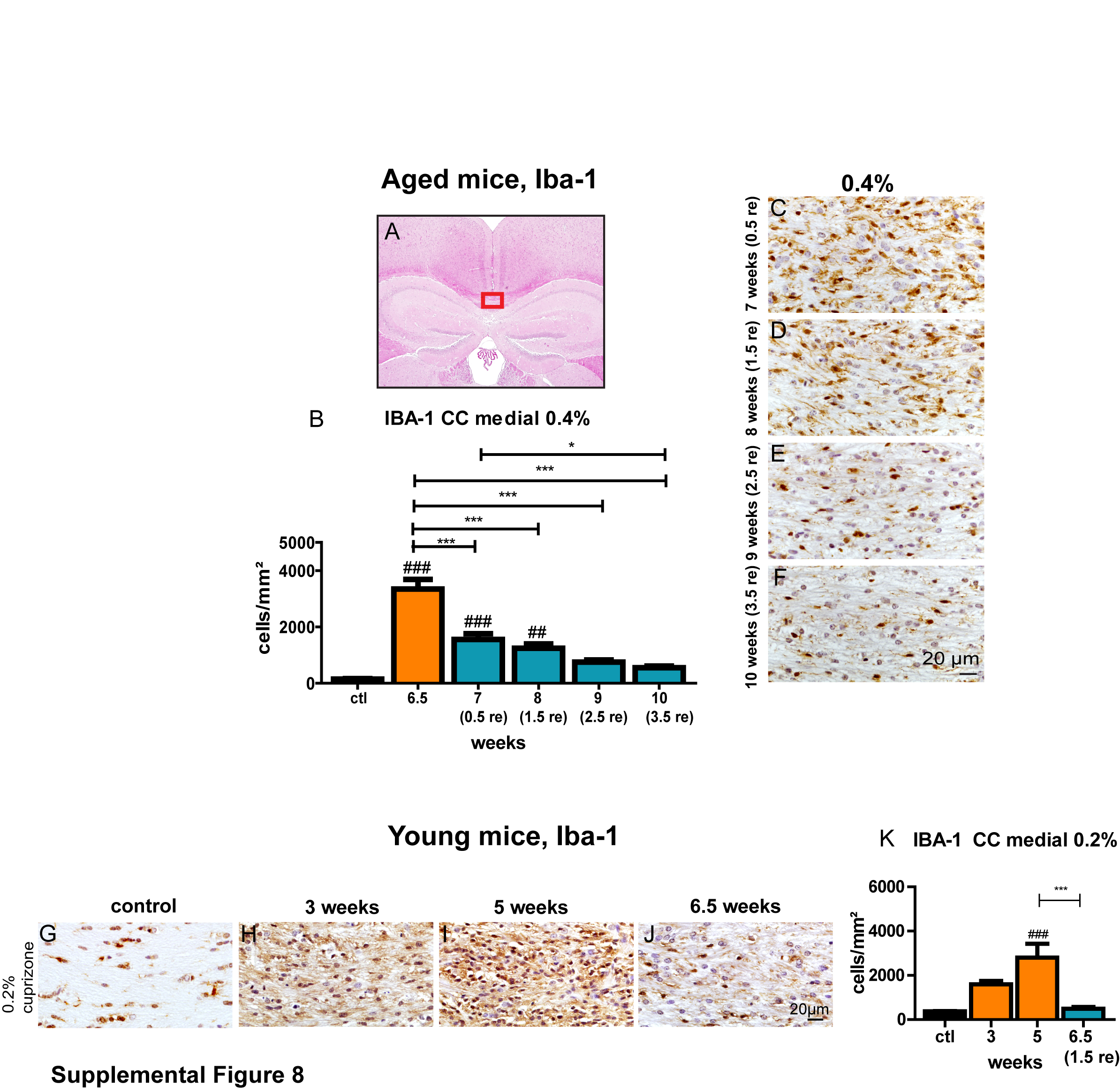

Supplement: Supplementary file 1 [file cells-09-00945-s001.zip › Supl 8_Microglia_Iba-1.tif]

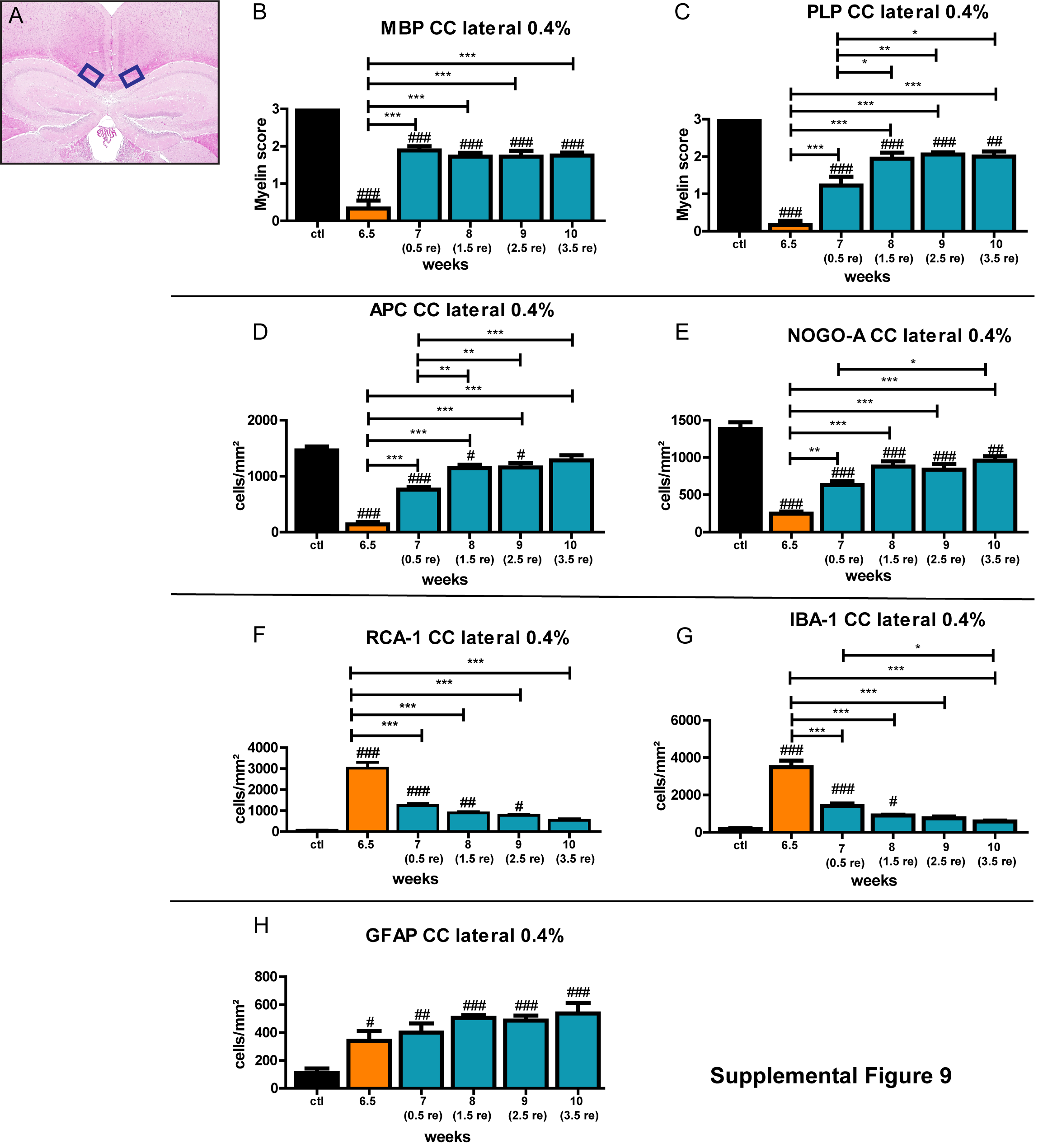

Supplement: Supplementary file 1 [file cells-09-00945-s001.zip › Supl 9_lateral_CC_remyelination.tif]

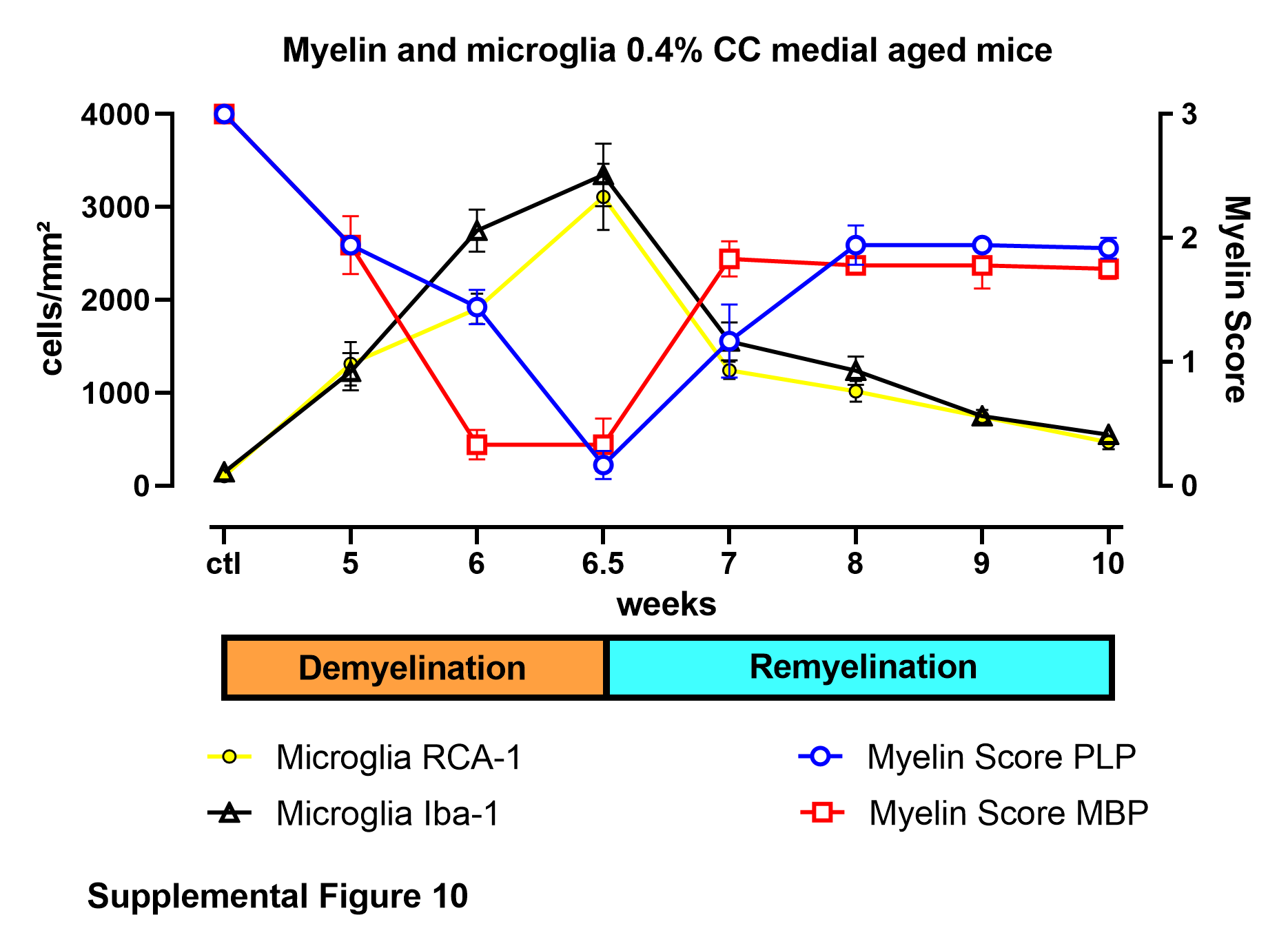

Supplement: Supplementary file 1 [file cells-09-00945-s001.zip › Supl 10_Myelin_and_Microglia.tif]
